# *Nkx3-1* and *Fech* genes might be switch genes involved in pituitary non-functioning adenoma invasiveness

**DOI:** 10.1038/s41598-021-00431-2

**Published:** 2021-10-22

**Authors:** Nasibeh Khayer, Maryam Jalessi, Amin Jahanbakhshi, Alireza Tabib khooei, Mehdi Mirzaie

**Affiliations:** 1 Skull Base Research Center, The Five Senses Health Institute, Iran University of Medical Sciences, Tehran, Iran; 2 ENT and Head & Neck Research Center and Department, Hazrat Rasoul Hospital, Iran University of Medical Sciences, Tehran, Iran; 3 Neurology Department, Hazrat Rasoul Hospital, Iran University of Medical Sciences, Tehran, Iran; 4 Department of Applied Mathematics, Faculty of Mathematical Sciences, Tarbiat Modares University, Tehran, Iran

**Keywords:** Cancer genomics, Biomarkers, Computational biology and bioinformatics, Data processing, Gene ontology, Gene regulatory networks, Statistical methods

## Abstract

Non-functioning pituitary adenomas (NFPAs) are typical pituitary macroadenomas in adults associated with increased mortality and morbidity. Although pituitary adenomas are commonly considered slow-growing benign brain tumors, numerous of them possess an invasive nature. Such tumors destroy sella turcica and invade the adjacent tissues such as the cavernous sinus and sphenoid sinus. In these cases, the most critical obstacle for complete surgical removal is the high risk of damaging adjacent vital structures. Therefore, the development of novel therapeutic strategies for either early diagnosis through biomarkers or medical therapies to reduce the recurrence rate of NFPAs is imperative. Identification of gene interactions has paved the way for decoding complex molecular mechanisms, including disease-related pathways, and identifying the most momentous genes involved in a specific disease. Currently, our knowledge of the invasion of the pituitary adenoma at the molecular level is not sufficient. The current study aimed to identify critical biomarkers and biological pathways associated with invasiveness in the NFPAs using a three-way interaction model for the first time. In the current study, the Liquid association method was applied to capture the statistically significant triplets involved in NFPAs invasiveness. Subsequently, Random Forest analysis was applied to select the most important switch genes. Finally, gene set enrichment (GSE) and gene regulatory network (GRN) analyses were applied to trace the biological relevance of the statistically significant triplets. The results of this study suggest that “mRNA processing” and “spindle organization” biological processes are important in NFAPs invasiveness. Specifically, our results suggest *Nkx3-1 and Fech* as two switch genes in NFAPs invasiveness that may be potential biomarkers or target genes in this pathology.

## Introduction

Pituitary adenomas (PAs) are the second most common primary brain tumors with substantial mortality rates^1,2^. PAs are categorized into non-functioning and functioning types based on clinical and biochemical features. Non-functioning pituitary adenomas (NFPAs) are the most common type of PAs in adults. In contrast with the functioning pituitary adenomas (FPAs), which release additional levels of endocrine hormones, NFPAs are not hormonally active^3^. The absence of any clinical and biochemical signs of hormone-excess leads to the late detection of NFPAs.

Furthermore, PAs are commonly considered slow-growing benign brain tumors, but a large number of them exhibit a local invasive behavior that is unpredictable with the aid of current tumor biomarkers^4^. The invasive PAs destroy sella turcica and invade the adjacent tissues such as the cavernous and sphenoid sinus. The most critical obstacle for total surgical removal is the high risk of involvement of adjacent vital nervous or vascular structures. On the other hand, despite technological improvements in surgical approaches and radiotherapy, the recurrence risk of invasive NFPAs remains high^5^. Therefore, the development of novel therapeutic strategies for early diagnosis as well as decreasing the recurrence rate of NFPAs is imperative. Hence, a comprehensive biological insight into the NFPAs invasiveness procedure is a primary step to achieve the above purpose.

High throughput gene expression data (i.e., the transcriptome) provide genome-scale snapshots of gene expression, rich sources of information for inferring gene relationships^6,7^. Identification of gene interactions has paved the way for decoding complex molecular mechanisms, including disease-related pathways and identifying the most momentous genes involved in a specific disease^8^. With the purpose of developing diagnostic and therapeutic strategies, several biomarkers and pathways have been reported associated with invasiveness in NFPAs through gene expression data analysis. Some of the most momentous potential biomarkers related to the aggressive nature of NFPAs are pituitary tumor transforming gene 1 (PTTG1)^9^, Ezrin (EZR)^10^, Ectoderm-Neural Cortex 1 (ENC1)^11^, WNT Inhibitory Factor 1(WIF1)^12^, E-cadherin (CDH1) and Neural cell adhesion molecule (NCAM)^13^. Moreover, previous studies identified a perturbation in some signaling pathways that can make NFPAs prone to invasiveness. The main reported pathways include the “WNT signaling pathway”^12^, “local suppression of the immune response pathway”, “TGF-β signaling”^14^, “PI3K-Akt signaling pathway” and “chemokine signaling pathway”^15^. However, notwithstanding that the molecular markers and pathways associated with NFPA invasiveness are extensively studied, much remains unknown.

Depending on applied mathematical and statistical methods, various gene expression patterns can be traced from the same biological dataset^16^. It should be noted that the above studies were done based on the two-way gene interaction approach. In the current study, we aimed to trace the three-way gene interaction pattern in the NFPA microarray gene expression dataset. We used the Liquid Association method^17^. The three-way gene interaction pattern draws the dynamic nature of the co-expression relation of two genes by proposing a third gene known as a switch gene^16^. Such a pattern deciphers the sophisticated molecular relations at a higher level than the conventional two-way gene interaction pattern, including co-expression^18,19^ and differentially co-expression^20^ patterns. Therefore, it can lead to a more comprehensive and explicit biological insight into the cause of cellular changes. The successful identification of the switch genes in diseases can be consequential because they can be regarded as potential drug targets. In the meantime, switching genes can be helpful in decoding biological complexities^21,22^.

The three-way gene interaction model is not investigated for NFPAs’ gene expression data to the best of our knowledge. The main challenge to implementing a three-way interaction model is presumably a large number of possible interactions for more than two genes at the genome-scale that result in a high computational load.

The current study aimed to identify critical biomarkers and biological pathways associated with invasiveness in the NFPAs, using the three-way interaction model. We hope that the results of this study provide efficient therapeutic targets and diagnostic or prognostic biomarkers.

## Results

### Determining statistically significant three-way interaction

**Figure 1:**
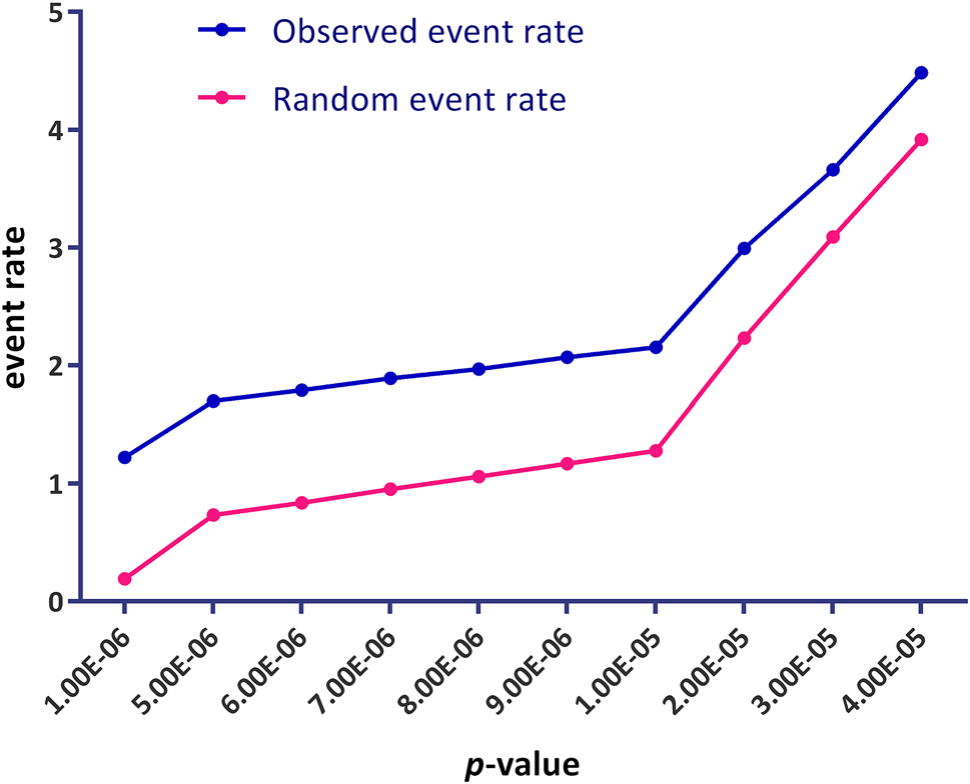
A survey of accuracy of fastLA analysis. In the wide range of the significant fastLA *p*-values, the observed event rate of X_3_ position (switch) genes was compared with the random event rate. The random event rate is equal to the ratio of the number of statistically significant triplets (in a particular p-value) to the number of total examination genes; the observed event rate is equal to the ratio of statistically significant triplets to the number of unique X3. $Random\, event\, rate= \frac{num.\, of\, significant\, triplets}{num.\, of\, totals\,examined\, genes}$; $Observed\, event\, rate= \frac{num.\, of\, significant\, triplets}{num.\, of\, unique\, {X}_{3}}$. As shown, the observed event rate of switch genes is significantly different from random, confirming the accuracy of fastLA analysis.

Using the fastLA package, liquid association analysis was performed for every combination of a candidate switching gene (X_3_) and every possible pair of genes ({X_1_, X_2_}) in the dataset. The top 200,000 triplets with the highest significance levels based on p-value were defined as outputs of this analysis. A *p*-value histogram of these three-way interactions is available in Fig. S1. To survey the validity of fastLA analysis, the observed event rate of X_3_ position (switch) genes was compared with random event rate in the wide range of the significant fastLA *p*-values. The plots of such comparison are presented in Fig. 1. Furthermore, changes in FDR using the Benjamini–Hochberg method versus − log (p-value) for the first 200,000 triplets are shown in the Fig. S2.

For the rest of our analysis, the set of all three-way interactions were chosen by considering FDR < 0.001 and, in addition, non-random observed rate in X_3_ position genes, consisting of 124 triple combinations. The list of all statistically significant triplets is presented in the Table S3.

### Gene selection using random forest

**Figure 2:**
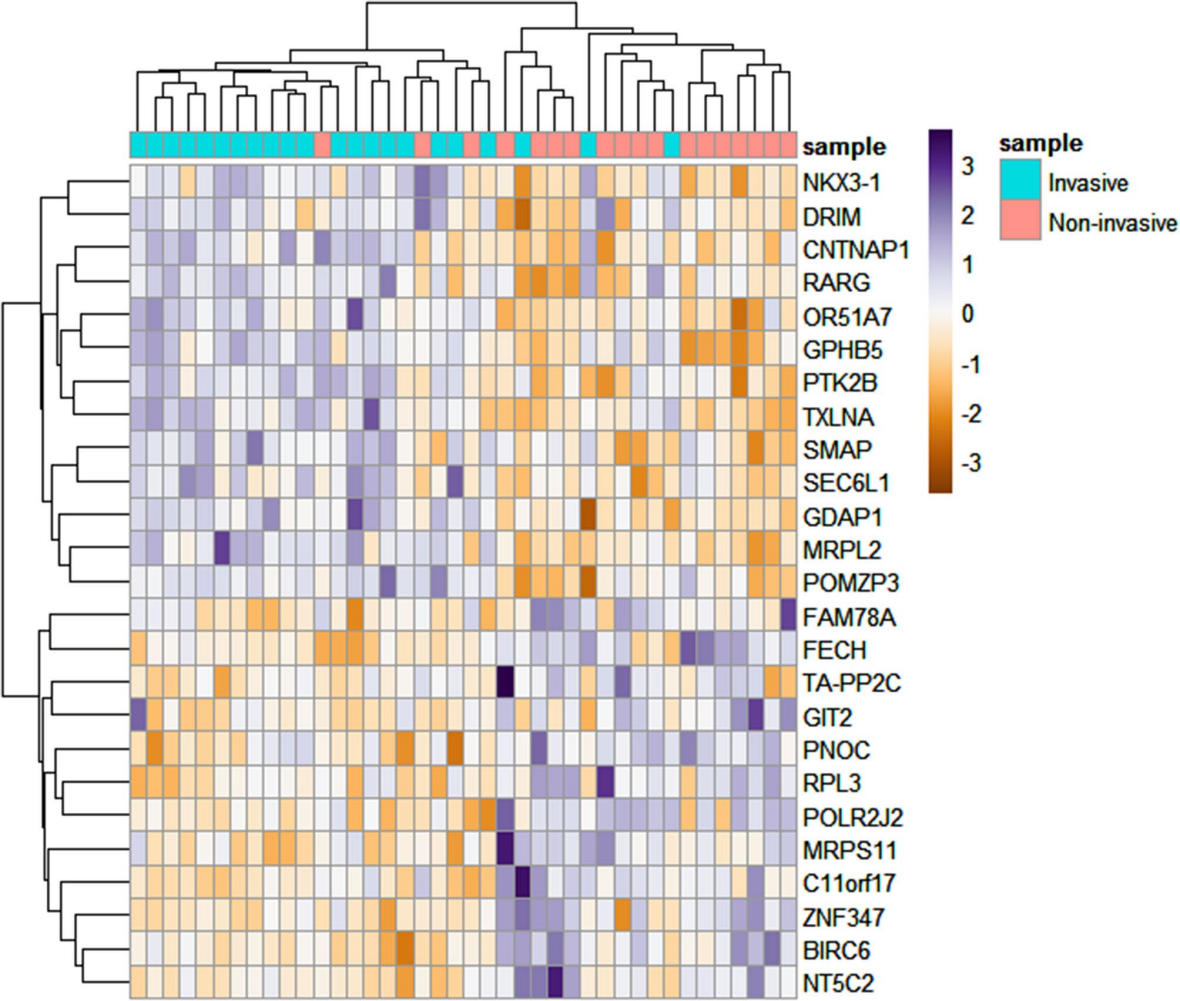
Random forest classification. This figure present 25 top importance genes selected based on Mean Decrease Accuracy measure.

Several measures of variable importance are obtained using the random forest algorithm. The most reliable measure is Mean Decrease Accuracy (MDA), which is based on the decrease of classification accuracy when the expression values of a particular gene are randomly permuted^23,24^. We reported 25 top importance genes selected based on MDA in Fig. 2. Furthermore, the area under the receiver operating characteristic (ROC) curve (AUC) is widely used as an assessment indicator to evaluate the performance of supervised classification models^25^. Therefore, ROC curves were used to analyze the sensitivity and specificity of the Random Forest model. As demonstrated in the Fig. S4, the AUC, sensitivity, and specificity of the classifier are 0.70, 67, and 82, respectively.

As continued, all 124 statistically significant triplets whose X_3_ position gene belongs to 25 top importance genes as well as the observed event rate of X_3_ position are far from random were selected to detect biologically relevant triplets.

### Identification of biologically-relevant triplets

**Figure 3:**
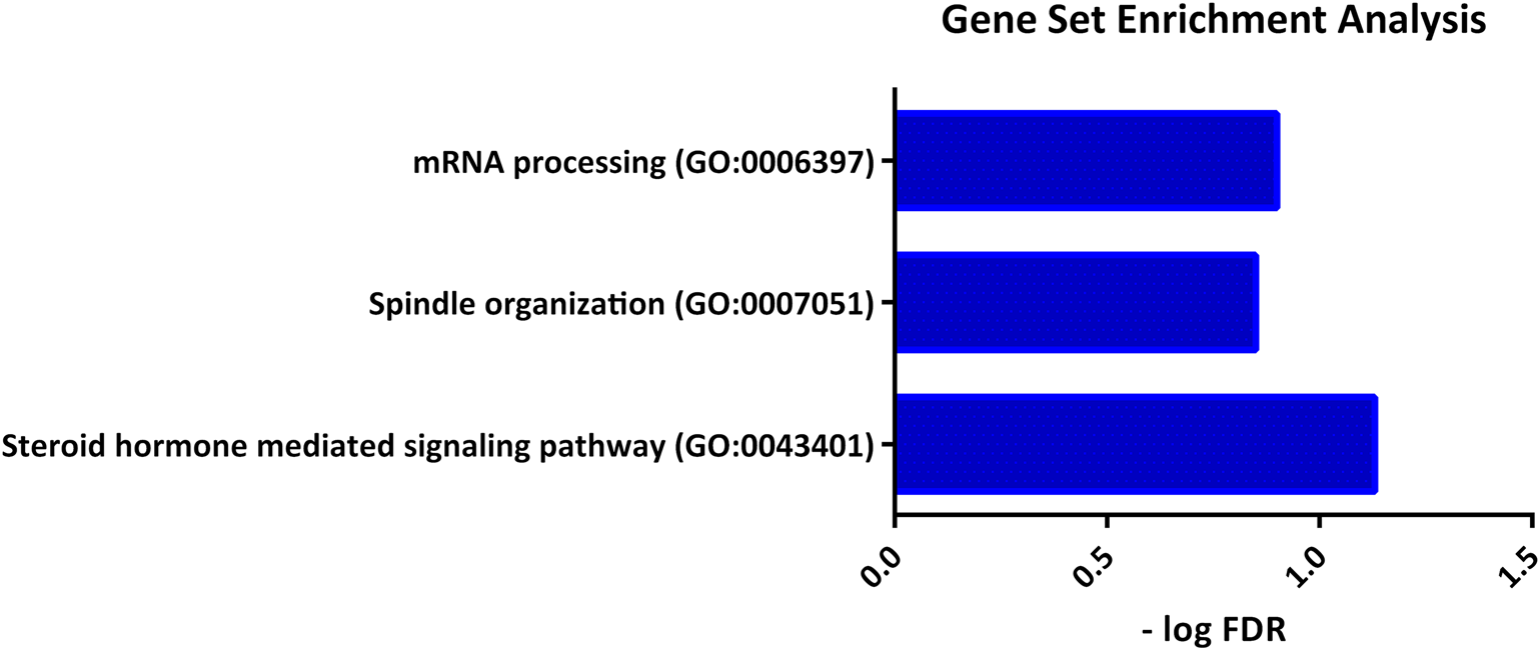
Biological process enrichment analysis. Enriched terms based on biological process for all genes involved in the statistically significant triplets.

We used GSEA in order to find biologically relevant triplets. Such analysis was performed using p-value < 0.05 and FDR < 0.1 for all of the involved genes in 124 statistically significant triplets (including 199 individual genes). Since the terms in lower levels of gene ontology are general, ones in levels lower than level 6 are not reported. As reported in Fig. 3, the enriched terms based on "biological process" as follows: “spindle organization”, “steroid hormone mediated signaling pathway” and “mRNA processing”. Based on the proposed definition of three-way interactions of the switch mechanism model, it is expected that in biologically relevant triplets, X_1_ and X_2_ are in the same biological process. The complete list of enriched terms is available in the Table S5.

By tracing triplets in enriched terms, three triplets in which X_1_ and X_2_ are involved in the same biological process were determined. Such triplets including *Nkx3-1*, {*Ckap5*, *Dlg1*} triplet, *Znf347*, {*Safb*, *Dnaja1*} triplet and *Fech*,{*Safb*, *Cdk9*} triplet that are involved in “spindle organization”, “steroid hormone mediated signaling pathway” and “mRNA processing”, respectively.

**Figure 4:**
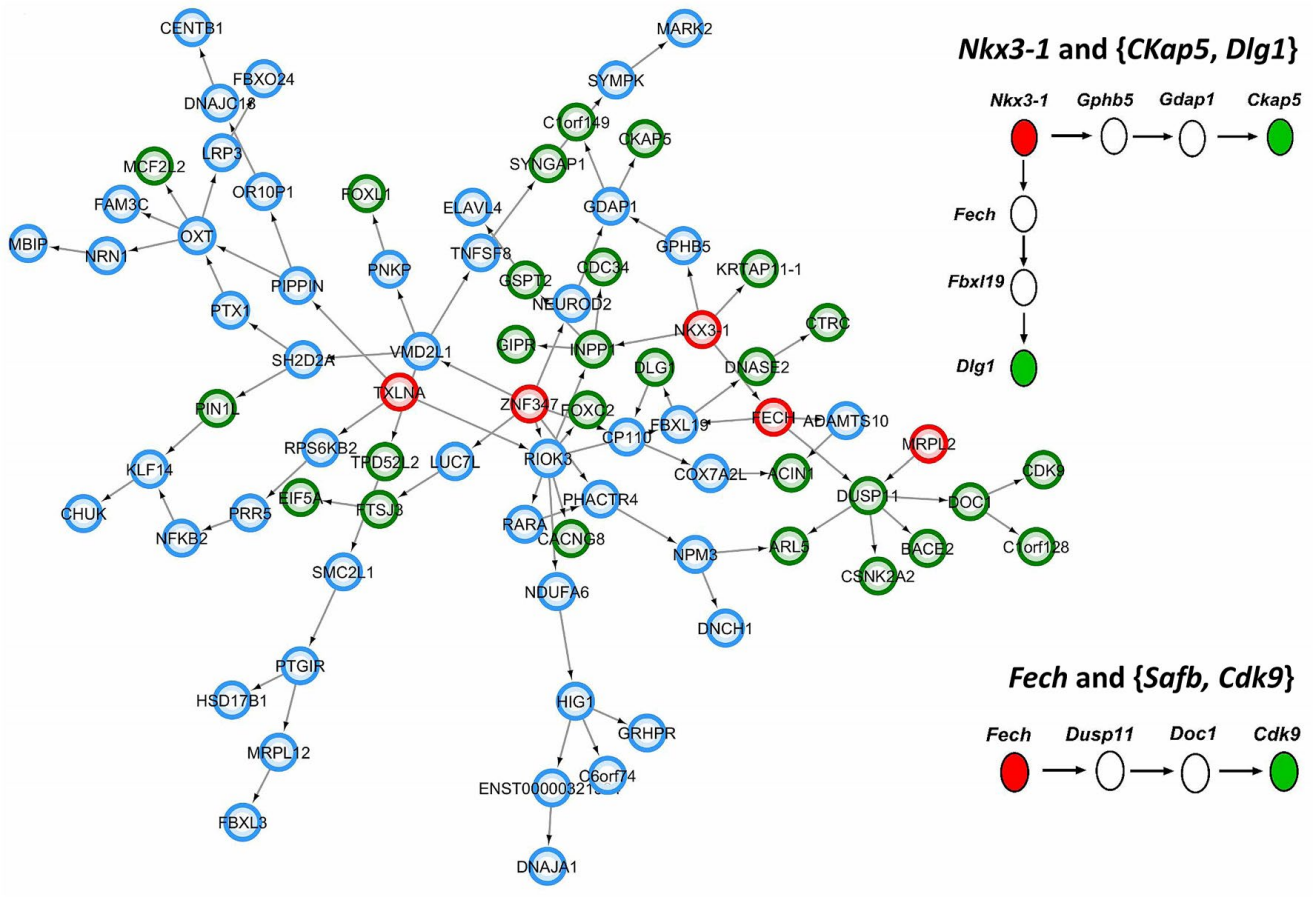
The position of biologically relevant triplets in Gene Regulatory Network (GRN). The biological relevance of thirty statistically significant triplets was confirmed GRN analysis. A subnetwork of GRN that includes the regulatory relations of such triplets is shown here. Red nodes represent the X_3_ position gene in each triplet, green nodes represent the X_1_ and X_2_ position genes, and other genes are presented by blue nodes.

As another attempt to analyze the functional relevance of three-way interactions, we reconstructed a GRN based on ARACNE. The regulatory relationship of significant triplets obtained from the liquid association method was traced in this network and the results are shown as a sub-network in Fig. 4. The details of construction GRN and detection of significant triplets in this network are available in the Table S6.

**Figure 5:**
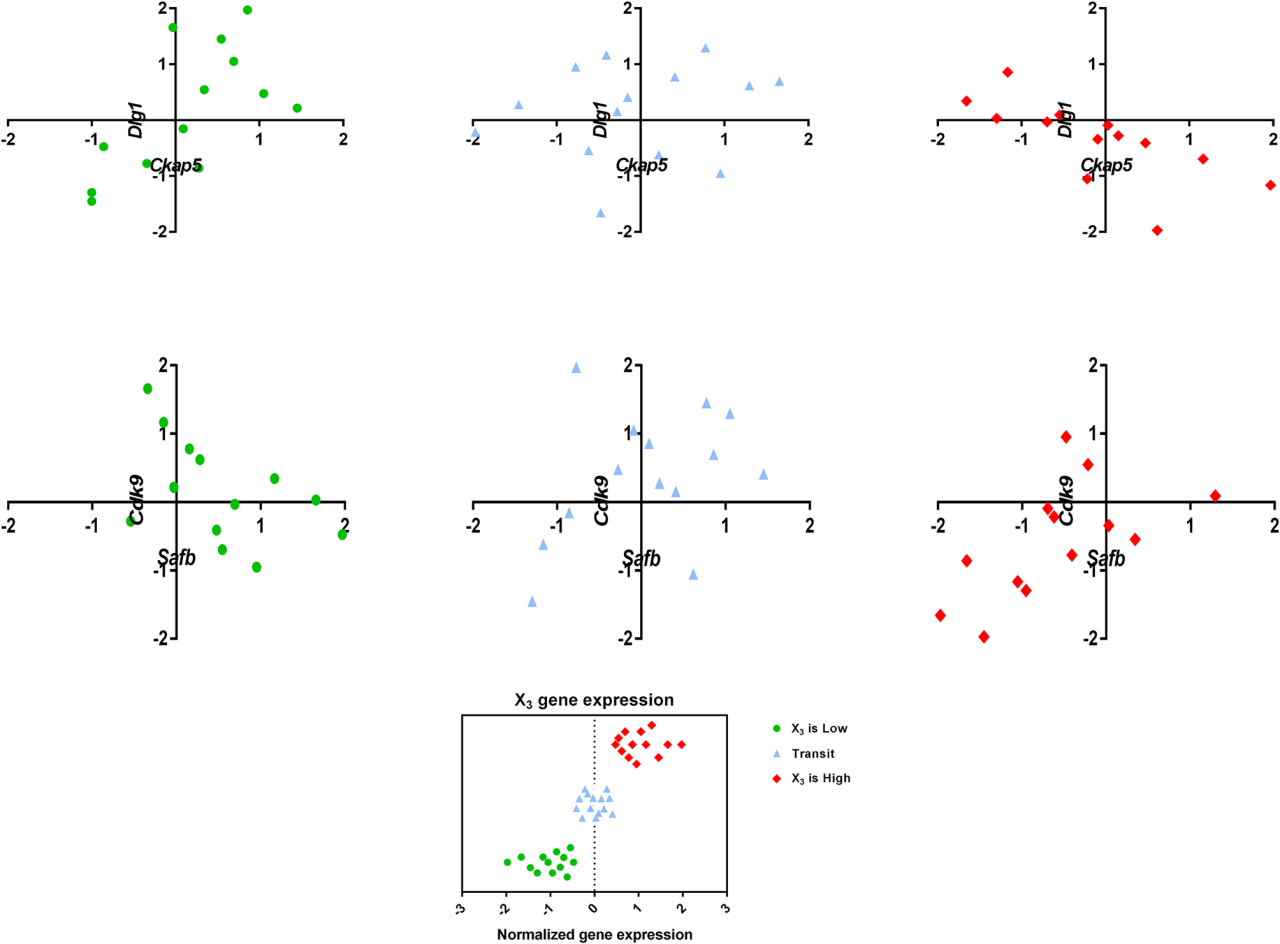
Scatter plot of two biologically relevant triplets. In each case, there is a considerable change in the correlation of X_1_ and X_2_ as a result of change in X_3_ expression level.

Conclusively, the biological relevancy of two statistically significant triplets was confirmed using both GSEA and GRN, including 22th and 46th triplets. The scatter plots of these triplets in three different ranges of associated X_3_ expression levels are shown in Fig. 5, which indicates a considerable change in the correlation of X_1_ and X_2_ as a result of a change in X_3_.

As it is observed, the regulatory relationship between X_3_ from the 22nd triplet (*Nkx3-1*) and two other genes in this triplet (*Ckap5* and *Dlg1*) can be seen with two intermediate genes. In addition, a regulatory interaction between X_3_ (*Fech*) and X_2_ (*Cdk9*) from the 46th triplet is observed in a nontrivial way.

**Figure 6:**
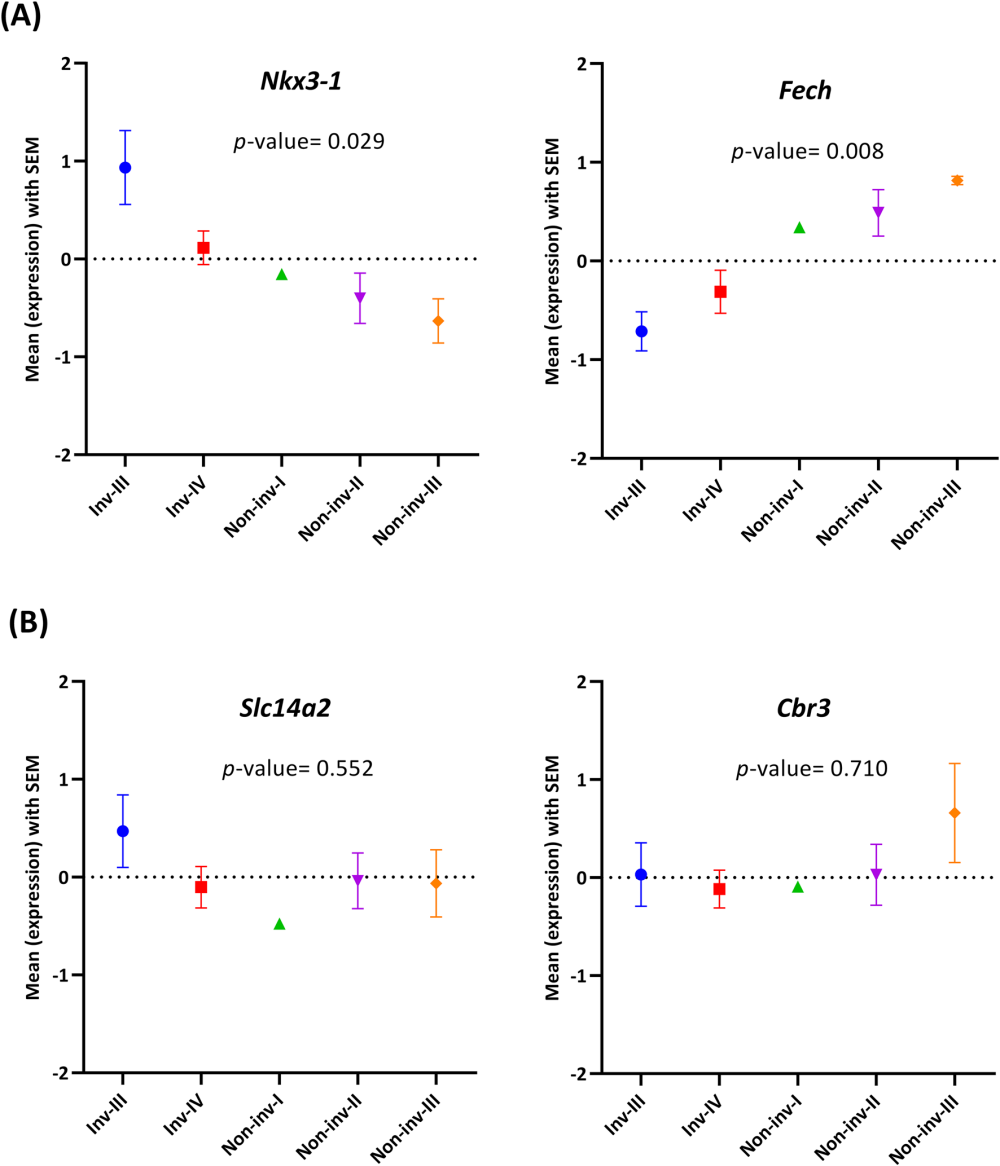
Mean expression level plot in the different grades of invasive and non-invasive pituitary adenomas for (**A**) two identified switch genes (i.e., Nkx3-1 and Fech genes); and (**B**) two exemplary random genes (i.e., Cl14a1 and Cbr3 genes).

To determine the possible associations of the identified switch genes with tumor grade, as the most important clinic-pathological feature, the mean expression level of two identified switch genes (i.e., *Nkx3-1* and *Fech* genes) were surveyed in the different grades of invasive and non-invasive pituitary adenomas. As shown in Fig. 6A, the samples correspond to each tumor grade were clearly separated based on the switch genes' expression level. Besides, to verify the accuracy of such a result, it was compared with ten randomly selected genes (Fig. 6B). The results show that the gene expression level of identified switch genes is correlated with tumor grade, but it is also significantly different from random. Such a feature is an advantage for any gene to be considered as a potential biomarker.

Figure 6B indicates two exemplary plots of mean expression levels of random genes in the various grades of NFPAs. Furthermore, additional information about all of the 15 randomly selected genes are presented in Fig. S7.

It should be noted that since the identified switch genes comprise approximately 0.1 percent of total surveyed genes (i.e., two from 2321 genes), we considered 15 random genes from the dataset, including 0.1 percent of all known genes in the dataset (ten from 15,215). The random genes were generated in the R environment.

Furthermore, the association of two identified switch genes with tumor volume, age at surgery, recurrence and gender were examined.

The results show that there might be an association between gene expression levels of both switch genes and tumors with volume greater than 20 cm^3^. Nevertheless, the number of samples corresponding to the aforementioned tumor is inadequate for such a conclusion (only three samples belong to the tumors greater than 20 cm^3^). Moreover, statistical analysis on the variance indicated significant changes in the expression levels of the *Fech* gene in the different groups of the recurrence feature (*p*-value = 0.016).

Finally, we did not find a considerable association between the expression levels of none of the switch genes and other clinic-pathological features, including gender and age at surgery.

The results of the above analyses were presented in the Fig. S8.

## Discussion

Although pituitary adenomas, including NFPAs, are commonly considered slow-growing benign brain tumors, a large number of them exhibit a local invasive behavior. Notwithstanding that transcriptome changes associated with NFPA invasiveness have been extensively studied in the NFPAs, which is unpredictable with the aid of current tumor biomarkers^4^. Therefore, the current study, for the first time, utilized the three-way interaction model to provide insights into upon the biological pathways as well as critical genes associated with invasive nature in the NFPAs.

The validity of fastLA analysis was confirmed by comparing the observed event rate of X_3_ position (switch) genes in a wide range of significant fastLA *p*-values and the random one. It is expected that the number of genes that occupy the X_3_ position be significantly lower than random because, as a biological concept, a limited number of genes controls most biological processes. As presented in Fig. 1, the observed event rate for switch genes is significantly different from random. Such a result means that certain genes occupy most X_3_ positions in the statistically significant triplets.

The biological relevancy of two statistically significant triplets was confirmed using both GSEA and GRN (see Figs. 3 and 4). Furthermore, the gene expression level of identified switch genes is significantly correlated with tumor grade (see Fig. 6). Such results suggest that these two triplets may play a central role in PA invasiveness.

In the following, we discussed the relationships between involved genes in such triplets separately.

### Relationship between involved genes in triplet *Fech*, {*Safb*, *Cdk9*}

In such triplet, *Fech* is the switch gene that controls the co-expression relationship between gene pair {*Safb*, *Cdk9*}. The protein encoded by the *Fech* gene is ferrochelatase, which is a crucial enzyme that catalyzes the conversion of protoporphyrin IX (PpIX) to heme. A significant down-regulation of *Fech* expression was found in several malignancies^26–29^, resulting in PpIX accumulation in such tumor cells. Indeed, accumulated PpIX in tumor cells leads to photodynamic therapy as effective adjuvant therapy for treating various cancers through visualizing the extent and margins of tumors, including PA^30^.

On the other hand, ferrochelatase is involved in endothelial cell growth and choroidal neovascularization^31^. Pusha and coworker^32^ found that inhibition of *Fech* reduces retinal neovascularization and endothelial cell proliferation in the oxygen-induced retinopathy (OIR) mouse model. Furthermore, they suggested griseofulvin as a Fech-inhibiting drug that could be repurposed to treat retinal neovascularization by blocking pathological tuft formation and revascularized areas of vaso-obliteration. Additionally, the inhibitory effect of griseofulvin is reported in skin carcinogenesis^33^, thyroid tumors^34^, and the development of multiple hepatomas^35^.

Although any direct effect of *Fech* gene expression on PA invasiveness is not reported until now, the positive association between gene expression of *Fech* gene and epidermal growth factor receptor (EGFR) as a critical gene involving in PA progression has been reported^36^.

The results of GSEA (see Fig. 3) show that such triplet is involved in the “mRNA processing” biological process. In the following, we discuss the importance of “mRNA processing” in PA invasiveness.

#### mRNA processing and PA invasiveness

Intrinsically, cancer evolves through successive genetic alterations that are advantageous to tumor cells. DNA sequence perturbations, as well as epigenomic disruption, are two significant cancer-related alterations^37^. However, besides the genetic changes, abnormalities in the mRNA processing can also trigger cancer formation and motive tumor progression^38^. Indeed, the mRNA processing known as a post-transcriptional mechanism is a crucial biological process during which pre-mRNA undergoes a series of chemical modifications to form the mature mRNA. Subsequently, mature mRNA can be transported to the cytoplasm and translated into the corresponding protein. Such biological processes comprise three critical steps: removing introns by splicing, cleavage the 3′end of mRNA, and polyadenylation^39^. Approximately forty years after recognizing the RNA processing, it is clear that post-transcriptional mechanisms are disrupted in cancer biology^40,41^. In other words, mRNA processing is frequently altered in the tumors. These alterations lead to the formation of numerous cancer-specific mRNAs translated to misfunction proteins and/or proteins with changed expression levels. Such changed proteins can result in the activation of oncogenes or the inactivation of tumor-suppressor genes^42,43^.

Moreover, abnormality in mRNA processing can be associated with cancer therapeutic resistance. Pre-mRNA processing factor 4 (PRPF4) is known as a novel therapeutic target for breast cancer treatment. The PRPF4 gene was overexpressed in various breast cancer cell lines. The PRPF4 gene was overexpressed in various breast cancer cell lines. Furthermore, Knockdown of the PRPF4 gene reduced migration and breast cancer invasion via suppressing the p38 MAPK phosphorylation pathway^44^. On the other hand, heterogeneous ribonucleoproteins (hnRNPs) that participate in different steps of pre-mRNA processing are involved in human malignancies and metastasis. Many reports also suggested several hnRNAs as promising therapeutic targets in numerous metastatic cancer types^45^. Furthermore, ubiquitin-specific peptidase 39 (USP39) serves critical roles in mRNA processing^46^ and additionally is involved in tumorigenesis of multiple solid malignancies^47,48^, including human renal cell carcinomas (RCC)^49^. Xu et al.^49^ show that silencing of USP39 by siRNA induced cell apoptosis and decreased invasive capacity of RCC cells. Hence, they suggested USP39 as an oncogenic factor that can play a pivotal role in human RCC treatment. Moreover, pre-mRNA processing factor (PRPF) 4B kinase^50^, pre-mRNA processing factor 19 (PRP19)^51,52^, and pre-mRNA processing factor 31 (PRP31)^53^ are the other oncogenic factors that are involved in mRNA processing pathway. Furthermore, the central role of the factors mentioned above is reported in previous studies in invasiveness and metastatic of numerous malignancies, including prostate cancer, melanoma, hepatocellular carcinoma, and invasive ovarian cancer.

To the best of our knowledge, there is no direct report on the role of the “mRNA processing” pathway in PA so far. Nevertheless, according to the above studies, there is considerable evidence to support that such a biological process may be associated with the invasiveness of PA.

### Relationship between involved genes in triplet *Nkx3-1*, {*Ckap5, Dlg1*}

The other significant triplet is *Nkx3-1* as the switch gene that controls the co-expression relationship between the gene pair {*Ckap5*, *Dlg1*}. The switch gene (*Nkx3-1*) is a homeodomain transcription factor with tumor suppressor function^54^. Homeobox genes comprise a large family of developmental regulators that are essential for cell differentiation and are often aberrantly expressed in cancer^55^. Furthermore, the *Nkx3-1* gene is a marker for diagnosing metastatic tumors^56,57^; besides, loss of *Nkx3-1*expression occurs in the early tumorigenesis, suggesting such gene plays a role in malignant initiation^56^. Surprisingly, such evidence is consistent with the concept of disease-related-switch genes.

In specific, dysregulation of *Nkx3-1* is known as a biomarker for prostate cancer progression^57–59^. Hereupon, anti-NKX3-1 antibodies are used as a method for diagnosing metastatic prostatic adenocarcinomas. Nevertheless, previous studies reported that loss of *Nkx3.1* expression correlates with several other malignancies, including breast cancer^57^ and salivary duct carcinoma^60^.

To the best of our knowledge, no direct link was reported between the *Nkx3-1* gene and the PA, although there is an indirect association. The *Fgf-2* gene, which plays a central role in the angiogenesis of invasive PA^61–63^, is an upstream regulator of NKX genes^64,65^. Furthermore, the importance of *Fgf-2* was reported in human prostate cancer progression^63^. Therefore, it can be inferred that *Fgf-2* might control the angiogenesis procedure by regulating the gene expression level of *Nkx3-1*.

The other aim of the current study was to comprehensively characterize which biological processes may be involved in the invasiveness of PA.

As shown in Fig. 3, the above triplet is involved in the “spindle organization” biological process. We discussed such a biological process in PA invasiveness. See below.

#### Spindle organization and PA invasiveness

Another enriched biological process is “spindle organization”, which assists the arrangement, assembly, and disassembly of spindle components. The spindle, which belongs to cytoskeletal components, is composed of an array of microtubules and associated molecules that forms between opposite poles of a eukaryotic cell during DNA segregation. Accordingly, the spindle plays a pivotal role in separating duplicated chromosomes apart. Hereupon, the correctness of spindle organization and its associated molecules during cell division is crucial for cell fate determination, tissue organization, and cell development. On the other hand, deregulation of cytoskeletal components is associated with several oncogenic phenotypes, including increased migration and invasion of cancer cells^66–68^.

Nucleolar and spindle-associated protein 1 (NUSAP1), a microtubule-binding protein, is selectively expressed in proliferating cells. Moreover, it plays a critical role in spindle microtubule organization^69^. The expression levels of NUSAP1 are increased in the G2 to mitosis transition and then immediately decreased after cell division^70^. Previous studies have reported that dysregulation of NUSAP1 is associated with invasion, proliferation, and migration in several malignancies^71–78^, including pituitary adenomas^79^. Additionally, Lee et al.^79^ showed that the *NUSAP1* gene upregulated in 95% of patients with pituitary adenomas using the qRT-PCR technique. On the other hand, a pyrrolopyrimidine-based microtubule-depolymerizing agent (PP-13) reduces the metastatic dissemination of invasive cancer cells. PP-13, through binding to the colchicine site of β-tubulin, disturbs microtubules’ organization; and consequently induces spindle multipolarity, mitotic cell cycle blockade, and apoptosis^80^. Moreover, Gilson et al.^81^ illustrated that low concentration PP-13 (130 nmol L^−1^) treatment significantly decreased the metastatic invasiveness of human cancer cells. Furthermore, they suggested that PP-13 might be a potential alternative to standard chemotherapy in drug-refractory tumors.

The “spindle organization” is defined as a child term for the “cell cycle” process according to the Gene Ontology Databank categories^82^. Several studies confirmed the significant role of the “cell cycle” in PA invasion and migration. See below.

Zhang et al.^83^ compared differentially expressed microRNAs (DEMs) in the invasive and non-invasive PA. They report that DEMs were significantly associated with the “cell proliferation” and “cell cycle” pathways. On the other hand, Zheng et al.^84^ showed that MiR-106b is upregulated in the invasive PA patients compare to non-invasive ones, associated with migration and invasion of pituitary adenoma cells. Moreover, they illustrated the inhibition of miR-106b remarkably suppressed proliferation and migration through the arrest of cell cycles. Some other biological molecules that can affect migration and invasion of PA through disturbing the “cell cycle” process include S100 calcium-binding protein A9^85^, cyclin B1^86^, Lactate dehydrogenase A^87^.

Altogether, the above evidence confirms the significant role of “spindle organization” in the invasiveness and migration of tumor cells.

### A comparison of results with the initial study

Galland et al.^4^ performed genome-wide expression analysis using microarray technology to determine possible biomarkers in NFPAs invasiveness. Although their study only focused on differentially expressed genes and the correlation among such genes was not studied, parts of their results are comparable with our study. See below.

With the purpose of identifying a specific gene expression profile in the invasive NFPAs, Galland et al. traced DEGs between invasive and non-invasive tumors. Furthermore, they verified the gene expression levels of the top 44 DEGs using qRT-PCR. Consequently, the overexpression of four genes, namely *Igfbp5*, *Myo5a*, *Flt3* and *Nfe2l1*, was confirmed.

A major part of DEGs, which were reported in the Galland et al. study, have coverage our results. More precisely, among them, 36 genes, including *Myo5a*, *Flt3* and *Nfe2l1*, were found in common across our results. Nevertheless, two identified switch genes (i.e., the *Fech* and *Nkx3-1* genes) were not found among the top 44 DEGs. There might be two possible explanations for such observation: (i) although DEGs play a critical role in switch mechanisms, there is not necessarily a direct relation between DEG significance and switch gene importance. In other words, a gene may be significantly expressed between two conditions (e.g., invasive and non-invasive samples), but it does not act as a considerable switch gene; (ii) a small proportion of DEGs were verified using the qRT-PCR technique in Galland et al. study. It is possible that if more genes had been examined, the two switch genes found in our study were also included.

Moreover, Galland et al. reported that the DEGs have known molecular functions such as “cell cycling and cell death”, “cellular assembly”, “morphology and motility” and “gene expression regulation”. Surprisingly, “cell cycling” and “cellular assembly” functions support the “spindle organization” biological process that was indicated in our GSEA results.

## Conclusion and further work

The existence of a considerable number of disease-related high-throughput “omics” datasets has provided studies about disease-related pathways and genes. In the current study, for the first time, we used the three-way interaction model to identify critical biomarkers and biological pathways associated with invasiveness in the NFPAs. The main advantage of such an approach compared to the pairwise co-expression approach is that the three-way interaction model can cope with the dynamic nature of co-expression relations by introducing a third gene known as the switch gene. Therefore, the three-way interaction model can lead to a more comprehensive and precise understanding of the cause of cellular changes. The switch genes can be considered potential drug targets; therefore, the successful identification of them in a disease can be momentous. More specifically, in the present study, we identified two triplets associated with the invasive nature of NFPAs; consequently, we suggested their corresponding switch gene (i.e., Fech and Nkx3-1 genes) as drug targets for invasive NFPAs. Moreover, we introduced two biological processes, “mRNA processing” and “spindle organization”, which might play a central role in the NFPAs invasiveness.

Although our study provides new insight into the invasive nature of NFPAs using computational methods, more efforts should be performed to validate such findings. A reasonable way to in-silico validation of our results is verifying them in other NFPAs' datasets. However, some crucial prerequisites should be considered for selecting a reliable dataset. The most critical prerequisite is an adequate sample size. The LA algorithm is based on correlation coefficient; on the other hand, the samples are divided into at least three bins during the LA analysis procedure. Since the statistical significance of the correlation coefficient is related to the sample size, such a parameter should be considered in choosing a decent dataset. Another significant prerequisite is the association between the samples of datasets following two approaches: (i) features, which in turn is related to the design similarity of corresponding studies; (ii) gene expression profile, which in turn, can be affected by variation across platforms used to generate data^88^.

In order to perform in-silico validation, three well-known omics databases, including ArrayExpress^89^, Gene Expression Omnibus (GEO)^90^ and The Cancer Genome Atlas (TCGA)^91^, were explored to find genomics and/or transcriptomics datasets associated with PA invasiveness. Unfortunately, the NFPAs dataset is scarce in the publicly available database. Consequently, no dataset was found that meets all of the above requirements. The scarcity of omics NFPAs dataset indicates the urgent need for further efforts on such data, which in turn pave the way for a better understanding of this disease.

In the next step, the relationship between the *Fech* gene and the {*Cdk9*,*Safb*} gene pair as well as the *Nkx3-1* gene and the {*Ckap5*,*Dlg1*} gene pair need to be experimentally validated.

## Materials and methods

### Gene expression profiling dataset

The selected dataset includes gene expression of 22 invasive and 18 non-invasive NFPA (with no hormone secretion), which is available at the Array Express database^89^ under accession number E-TABM-899^4^. Additionally, it was generated using the A-AGIL-11- Agilent Human Whole-genome microarray platform. The samples belong to grade I to III of non-invasive NFPAs and grade III and IV of invasive NFPAs. The related clinical data of invasive and non-invasive PA samples were reported in Table S9. Additionally, detailed information about tumor size and grade selection criteria is found in Galland and co-worker's study^4^.

The background correction on the raw microarray dataset was carried out using the Normex method^92^. Furthermore, the expression profiles were normalized within- and between—arrays using loess^93^ and quantile normalization^94^ methods, respectively. It should be noted that the above-mentioned methods were implemented in the Limma R package^95^.

Moreover, the duplicate probes were removed using the genefilter package^96^. Accordingly, the highest interquartile range (IQR) across probes corresponds to each gene is retained. Furthermore, unchanged genes were removed from the microarray dataset because they do not provide valuable information to decipher gene expression relationships. For this purpose, the empirical Bayes method^97^ was used to detect differentially expressed genes (DEGs). Additionally, the Benjamini–Hochberg method^98^ was used to control the false discovery rate. After removing duplicated probes as well as probes corresponding to unknown genes, the dataset included 15,215 genes. Furthermore, by considering *p*-value < 0.01 as the threshold, DEGs include 2321 genes that were selected for further consideration.

Moreover, to assess the association of identified switch genes with clinic-pathological features, a statistical analysis on the variance was performed using Kruskal–Wallis ANOVA^99^. A *p*-value of less than 0.05 was considered statistically significant. Data are presented as means ± SEM.

### Liquid association triplets

Three-way interactions between all genes involved in the dataset were calculated using the fastMLA function in the fastMLA R package^100^. This package uses a modified liquid association algorithm for determining changes in coexpression relations of a gene pair, X_1_ and X_2_, based on the expression level of a third gene (X_3_).

Indeed, the fast modified liquid association algorithm computes an MLA score for each gene triplet to assess the magnitude of the liquid association. More specifically, MLA (X_1_, X_2_ |X_3_) can be estimated as:

$$\widehat{MLA} = \frac{{\sum\limits_{i}^{M} {\widehat{{\rho_{i} }}} \overline{{X_{3i} }} }}{M}$$

where *M* is the number of bins over X_3_, $\widehat{{\rho_{i} }}$ is the Pearson’s correlation coefficient of X_1_ and X_2_ in samples of the *i*th bin, and $\overline{{X_{3i} }}$ is the mean of expression values of X_3_ in the *i*th bin.

It should be not that before running fastMLA, performing two preprocessing steps are required: (i) to reduce the number of potential outliers in the data, the marginal distribution of each variable should be normal. Therefore, a normal quantile transformation was performed based on Li's approach^101^; (ii) each variable should be standardized to have mean 0 and variance 1^17^. The first preprocessing was performed using an in-house implementation, while the second one by using the CTT package^102^.

False discovery rate (FDR) was estimated using the Benjamini–Hochberg correction method, and liquid association triplets with FDR < 0.001 were chosen as statistically significant triplets. Subsequently, all triplets with the non-random observed rate in X_3_ position genes were retained for further study.

### Random Forests clustering

Random Forest (RF) is a powerful ensemble algorithm based on machine learning. Such an algorithm generates a collection of decision trees that are learned independently by bootstrap sampling. Each tree recursively divides observations into more homogeneous subsets. Finally, the outcome is obtained by combining a collection of accurately chosen classification trees.

A random forests classifier was built using the randomForest R package^23^. The “number of decision trees” and “mtry” parameters were set to “10,000 trees” and “square root of the total number of features”, respectively^103^. Finally, the gene importance measure is computed by averaging the increase in the error rate over all the trees.

### Pathway and functional enrichment analysis

Functional enrichment analysis is utilized to ascertain biologically relevant triplets and determine the central pathways and biological processes involved in PA. Functional enrichment analysis is a statistical method to classify genes (proteins) over-presented in a particular dataset using predefined annotations^104,105^. For all of the genes involved in all statistically significant triplets, functional enrichment analysis was performed based on the biological process using the gene ontology (GO) database. Furthermore, the same analyses were performed to find enriched pathways in the KEGG database^106^. For the analyses, as mentioned earlier, we used the ClueGO tool^107^ (with a Kappa threshold of 0.4) within the Cytoscape v.3.3.0 environment^108^. The right-sided hypergeometric test and the Benjamini–Hochberg correction method^109^ were used to validate enrichment analysis. Subsequently, comparing the enriched GO terms and KEGG pathways was performed to recognize the different biological processes between the invasive and non-invasive samples.

### Gene regulatory network construction

A gene regulatory network (GRN) models complex regulatory mechanisms that control the gene expression levels of mRNA, which, in turn, govern the function of the cell. A GRN consists of nodes (genes) and edges (regulatory relations) that can help to predict changes in gene expression under different conditions^110^. Here, we used ARACNE (Algorithm for the Reconstruction of Accurate Cellular Networks)^111^ to construct the GRN. ARACNE is a reverse engineering approach for the construction of cellular networks from gene expression data. This algorithm capture directed regulatory interactions between each transcriptional regulator and its potential targets based on mutual information. ARACNE run in the geWorkbench_2.6.0 framework for all of the genes involved in the statistically significant triplets by considering *p*-value < 0.05.

## Supplementary Information

Supplementary Information.

## Data Availability

The authors confirm that the data supporting the findings of this study are available within the article and its supplementary materials.
