# Publisher Correction: Nkx3-1 and Fech genes might be switch genes involved in pituitary non-functioning adenoma invasiveness

**DOI:** 10.1038/s41598-021-03094-1

**Published:** 2021-12-14

**Authors:** Nasibeh Khayer, Maryam Jalessi, Amin Jahanbakhshi, Alireza Tabib khooei, Mehdi Mirzaie

**Affiliations:** 1 Skull Base Research Center, The Five Senses Health Institute, Iran University of Medical Sciences, Tehran, Iran; 2 ENT and Head & Neck Research Center and Department, Hazrat Rasoul Hospital, Iran University of Medical Sciences, Tehran, Iran; 3 Neurology Department, Hazrat Rasoul Hospital, Iran University of Medical Sciences, Tehran, Iran; 4 Department of Applied Mathematics, Faculty of Mathematical Sciences, Tarbiat Modares University, Tehran, Iran

Correction to: *Scientific Reports* 10.1038/s41598-021-00431-2, published online 22 October 2021

In the original version of this Article, Maryam Jalessi was omitted as a corresponding author. Correspondence and requests for materials should also be addressed to jalessi.m@iums.ac.ir.

