# Supplementary material for: Nkx3-1 and Fech genes might be switch genes involved in pituitary non-functioning adenoma invasiveness
Source: Sci Rep. 2021 Oct 22;11:20943. doi: 10.1038/s41598-021-00431-2 (PMC8536755; doi:10.1038/s41598-021-00431-2)
Supplement: Supplementary file 1 — Supplementary Information. [file 41598_2021_431_MOESM1_ESM.pdf]

Supplementary Files:

S1 Fig. The *p*-value histogram of the top 200000 three-way interactions.

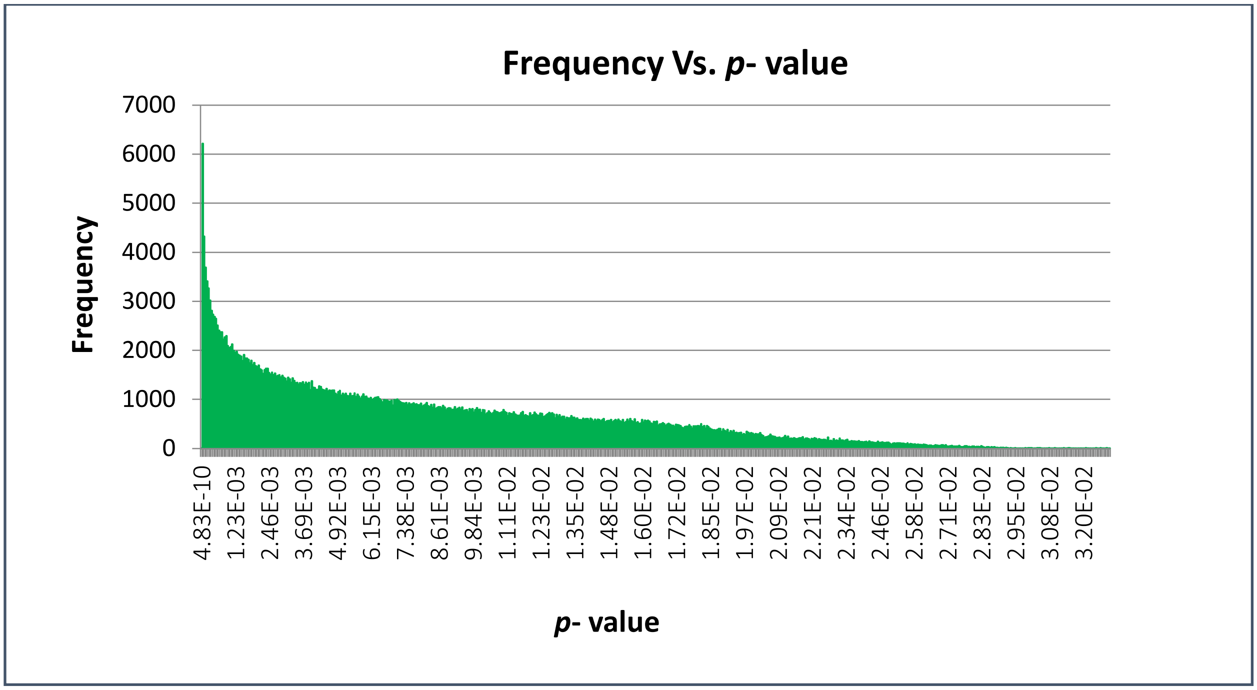

S2 Fig. FDR vs. -log (*p*-value). The changes in FDR (BH-corrected p-value) versus -log (p-value) for the first 200000 results of fastLA. As shown, FDR = 0.001 corresponds to -log (*p*-value) = 4.1.

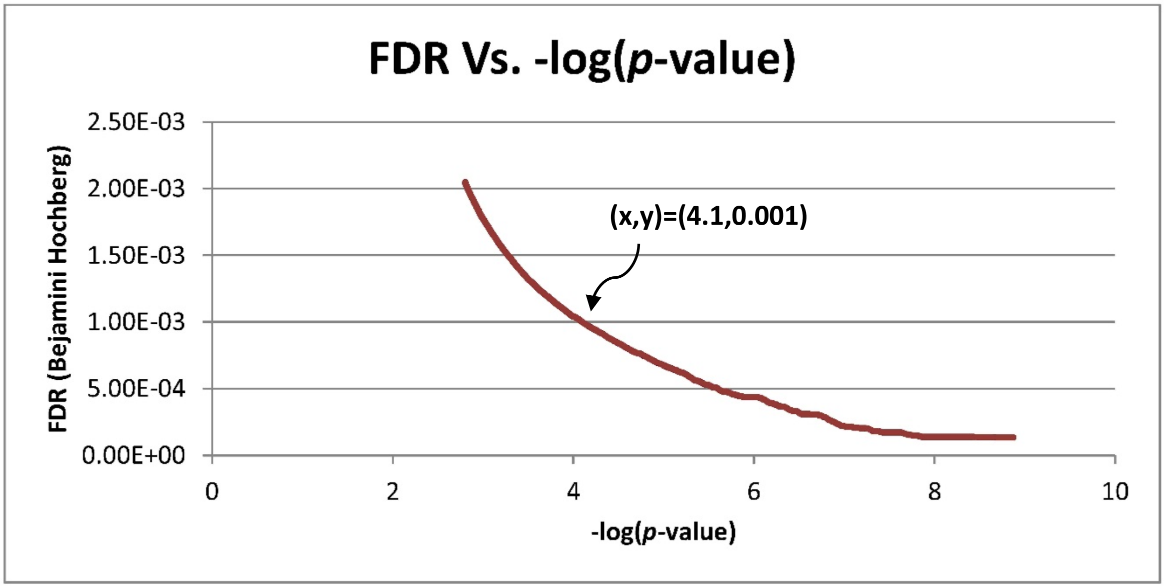

S3 Table. The list of 124 statistically significant triplets**.**

NUM	X1.or.X2	X2.or.X1	X3	rhodiff	MLA.value	wald	p.value	BH
1	FTSJ3	CACNG8	FECH	1.5266	0.5633	25.851	3.69E-07	3.33E-04
2	ZNF575	OR10P1	FECH	1.1581	0.4665	25.8163	3.76E-07	3.34E-04
3	HRK	GNPDA1	MRPL12	1.2804	0.4829	23.9737	9.77E-07	4.37E-04
4	IER3IP1	ARL5	MRPL2	1.351	0.4781	23.7368	1.10E-06	4.37E-04
5	LUC7L	POLR1A	FECH	1.2345	0.4945	23.4888	1.26E-06	4.37E-04
6	ATP7B	SMC2L1	FECH	-1.2163	-0.4862	23.3172	1.37E-06	4.44E-04
7	CACNG8	ENST00000326382	MRPL2	-1.2971	-0.4818	21.8041	3.02E-06	5.19E-04
8	NDUFA6	CRAT	NKX3-1	-1.4728	-0.5539	20.9204	4.79E-06	5.75E-04
9	UBE1C	WNT10B	MRPL2	1.271	0.4705	20.5064	5.94E-06	6.15E-04
10	INPP1	EFNA2	FECH	-1.2528	-0.5152	20.4995	5.96E-06	6.16E-04
11	RBMX2	MCF2L2	TXLNA	-1.2472	-0.51	20.3224	6.54E-06	6.24E-04
12	KENAE	PNKP	MRPL2	-1.4399	-0.4757	20.1102	7.31E-06	6.42E-04
13	MRFAP1	RORC	MRPL12	1.4209	0.466	19.844	8.40E-06	6.56E-04
14	RBMX2	SOCS1	MRPL12	1.2637	0.4825	19.8366	8.44E-06	6.56E-04
15	DNAJC18	ADRB3	GDAP1	1.5438	0.4808	19.7773	8.70E-06	6.58E-04
16	GIPR	ATP6V0D1	NKX3-1	-1.404	-0.503	19.7281	8.93E-06	6.60E-04
17	ESPL1	PTGIR	FECH	1.2659	0.4819	19.6994	9.06E-06	6.62E-04
18	RPS10	CTRC	FECH	-1.1493	-0.4849	19.5779	9.66E-06	6.71E-04
19	DUSP11	SYMPK	NKX3-1	1.6033	0.4897	19.5639	9.73E-06	6.71E-04
20	T1	XPOT	GPHB5	1.0765	0.467	19.4647	1.02E-05	6.79E-04
21	TBC1D17	UPF2	GPHB5	1.1835	0.4828	19.1294	1.22E-05	7.01E-04
22	CKAP5	DLG1	NKX3-1	-1.3742	-0.4645	19.1018	1.24E-05	7.03E-04
23	THRAP4	NOL8	TXLNA	1.313	0.4698	19.0348	1.28E-05	7.08E-04
24	CKAP5	TCERG1	FECH	1.1366	0.5242	18.9346	1.35E-05	7.16E-04
25	FHIT	TPD52L2	MRPL2	1.4721	0.4834	18.8616	1.41E-05	7.23E-04
26	KIAA0319L	KLC2	GDAP1	-1.1428	-0.5058	18.7256	1.51E-05	7.35E-04
27	PPM1L	NOL8	TXLNA	1.3872	0.4846	18.5144	1.69E-05	7.50E-04
28	TRIM2	GPX2	GDAP1	1.132	0.5087	18.5116	1.69E-05	7.50E-04
29	ACIN1	ENST00000321394	ZNF347	-1.2998	-0.5205	18.489	1.71E-05	7.53E-04
30	RCC2	CP110	GPHB5	-1.3789	-0.4663	18.4283	1.76E-05	7.59E-04
31	LRP3	ARL5	MRPL2	-1.2475	-0.5006	18.3831	1.81E-05	7.64E-04
32	DOCK7	RTN4RL2	GPHB5	1.1779	0.4584	18.3573	1.83E-05	7.64E-04
33	TPD52L2	VPS26	ZNF347	1.1111	0.4599	18.3064	1.88E-05	7.66E-04
34	FTSJ3	NR5A2	FECH	0.995	0.4758	18.3	1.89E-05	7.66E-04
35	FBXL19	TPD52L2	MRPL2	1.3463	0.5312	18.2624	1.92E-05	7.67E-04
36	SAFB	DNAJA1	ZNF347	-1.5352	-0.4718	18.2039	1.98E-05	7.68E-04
37	TPD52L2	GPR153	MRPL2	1.3563	0.5207	18.183	2.01E-05	7.71E-04
38	RPS10	ZNF529	FECH	1.4387	0.5167	18.0337	2.17E-05	7.79E-04

39	X75962	SPTBN4	GDAP1	-1.0323	-0.4755	17.9711	2.24E-05	7.83E-04
40	CHUK	SYNGAP1	ZNF347	-0.8329	-0.462	17.9681	2.25E-05	7.84E-04
41	SP3	COX7A2L	FECH	-1.1035	-0.4814	17.8756	2.36E-05	7.93E-04
42	SAMD8	RIOK3	FECH	1.0567	0.4688	17.8645	2.37E-05	7.95E-04
43	KARS	CXCL3	NKX3-1	1.1819	0.5192	17.8247	2.42E-05	7.97E-04
44	ZNF415	C6orf74	GDAP1	-1.5143	-0.4765	17.6294	2.68E-05	8.12E-04
45	TPD52L2	FAM3C	ZNF347	1.0802	0.4679	17.6035	2.72E-05	8.16E-04
46	SAFB	CDK9	FECH	1.1063	0.4619	17.5749	2.76E-05	8.20E-04
47	C15orf16	CLDN14	GDAP1	-1.1344	-0.4767	17.5072	2.86E-05	8.27E-04
48	FBXO9	GSTO2	MRPL12	-1.6549	-0.471	17.4883	2.89E-05	8.28E-04
49	CACNG8	GSPT2	TXLNA	1.4397	0.4644	17.4748	2.91E-05	8.29E-04
50	NYX	ARL5	MRPL2	-1.4088	-0.4896	17.4477	2.95E-05	8.32E-04
51	VMD2L1	XRCC5	FECH	-1.2836	-0.472	17.4365	2.97E-05	8.32E-04
52	INPP1	MRPL49	TXLNA	1.0731	0.4638	17.4194	3.00E-05	8.34E-04
53	HDHD3	PPIE	NKX3-1	1.426	0.4775	17.4084	3.01E-05	8.35E-04
54	FTSJ3	EIF5A	ZNF347	1.2303	0.5167	17.362	3.09E-05	8.38E-04
55	NRN1	UQCRC1	FECH	1.2454	0.4738	17.2376	3.30E-05	8.47E-04
56	UFC1	UBAP1	FECH	1.2297	0.4623	17.2278	3.32E-05	8.49E-04
57	BBC3	TPD52L2	MRPL2	1.4908	0.5406	17.1826	3.40E-05	8.54E-04
58	TPD52L2	SH2D2A	MRPL2	1.3186	0.492	17.1647	3.43E-05	8.54E-04
59	TPD52L2	HES7	MRPL2	1.4465	0.5626	17.0469	3.65E-05	8.65E-04
60	FAM79A	ALDH3B1	GDAP1	1.5175	0.504	17.0261	3.69E-05	8.66E-04
61	ARL5	ZNF575	MRPL2	-1.247	-0.4854	16.9965	3.74E-05	8.69E-04
62	PRR5	TPD52L2	MRPL2	1.2664	0.5115	16.9495	3.84E-05	8.73E-04
63	LUC7L	ASTL	FECH	1.2353	0.4765	16.9455	3.85E-05	8.73E-04
64	NFKBIL2	C6orf74	TXLNA	1.3489	0.4607	16.9172	3.90E-05	8.75E-04
65	PIPPIN	FAH	FECH	1.1173	0.4624	16.9165	3.91E-05	8.75E-04
66	NCL	COX7A2L	FECH	-1.292	-0.4704	16.8873	3.97E-05	8.77E-04
67	PIN1L	ARL5	ZNF347	1.021	0.4873	16.8832	3.98E-05	8.77E-04
68	EMP3	UPF2	GPHB5	1.4366	0.5014	16.8632	4.02E-05	8.79E-04
69	ARL5	SPHK2	ZNF347	1.122	0.4834	16.8388	4.07E-05	8.81E-04
70	TEAD3	UPK1A	NKX3-1	-1.3628	-0.4989	16.8251	4.10E-05	8.82E-04
71	RPS10	NEUROD2	FECH	-1.3209	-0.5252	16.7934	4.17E-05	8.86E-04
72	FTSJ3	CLDN4	FECH	1.178	0.4741	16.7139	4.35E-05	8.94E-04
73	GRHPR	CDC34	ZNF347	-1.3321	-0.4627	16.7069	4.36E-05	8.95E-04
74	KRTAP11-1	RPS8	NKX3-1	1.1831	0.4832	16.677	4.43E-05	8.98E-04
75	ATP7B	RBBP9	MRPL12	1.0542	0.4679	16.6688	4.45E-05	8.99E-04
76	KARS	ZNF137	FECH	1.1084	0.4891	16.6562	4.48E-05	9.01E-04
77	HIG1	T1	GPHB5	0.919	0.4653	16.6252	4.55E-05	9.03E-04
78	NPM3	MORF4L1	MRPL12	1.3072	0.4454	16.5221	4.81E-05	9.14E-04
79	NFKB2	FTS	MRPL2	1.4602	0.5205	16.4869	4.90E-05	9.18E-04

80	UTX	ADAMTS10	MRPL2	1.0917	0.4714	16.4784	4.92E-05	9.18E-04
81	PCDHGA7	OR10P1	FECH	0.9506	0.4682	16.4185	5.08E-05	9.22E-04
82	KLF14	CENTB1	TXLNA	-1.3893	-0.499	16.3979	5.13E-05	9.23E-04
83	ACIN1	C1orf128	FECH	-1.2932	-0.4968	16.3863	5.17E-05	9.25E-04
84	DNASE2	ELAVL4	TXLNA	1.1765	0.4735	16.3707	5.21E-05	9.26E-04
85	FBN3	INPP1	FECH	-1.3659	-0.5219	16.3453	5.28E-05	9.28E-04
86	KRTAP2-4	TPD52L2	MRPL2	1.1505	0.4969	16.3118	5.37E-05	9.32E-04
87	RTN3	MPFL	GPHB5	-0.9702	-0.4927	16.299	5.41E-05	9.33E-04
88	SLC12A9	TNFSF8	MRPL12	-1.28	-0.449	16.2934	5.43E-05	9.34E-04
89	FBXL3	OR2T5	FECH	-1.3764	-0.5149	16.2925	5.43E-05	9.34E-04
90	ZNF668	UQCRC1	FECH	1.2655	0.4613	16.2634	5.51E-05	9.35E-04
91	FBXL3	OXT	MRPL2	1.1163	0.469	16.2608	5.52E-05	9.36E-04
92	C1orf149	FOXL1	ZNF347	1.2379	0.4787	16.2349	5.60E-05	9.39E-04
93	CDC42EP1	UPF2	GDAP1	1.5123	0.4838	16.2257	5.62E-05	9.40E-04
94	ARL5	NEUROG1	MRPL2	-1.2131	-0.4753	16.203	5.69E-05	9.43E-04
95	SPRN	LAPTM4A	NKX3-1	-1.2322	-0.4678	16.1659	5.80E-05	9.44E-04
96	MBIP	SLC38A5	ZNF347	-1.3164	-0.4891	16.1336	5.90E-05	9.47E-04
97	CSNK2A2	SF3B2	NKX3-1	1.062	0.4772	16.1076	5.98E-05	9.49E-04
98	SLC25A2	SMC2L1	FECH	-1.33	-0.4934	16.1036	6.00E-05	9.50E-04
99	RBMX2	FOXC2	TXLNA	1.2009	0.524	16.0995	6.01E-05	9.50E-04
100	RARA	FAM61B	FECH	1.1562	0.4636	16.0756	6.09E-05	9.51E-04
101	DNAJC18	CTRC	FECH	-1.0692	-0.4793	16.0492	6.17E-05	9.54E-04
102	PTX1	SIL	FECH	1.2964	0.4862	16.0456	6.18E-05	9.54E-04
103	KCNS2	CEPT1	MRPL2	1.1408	0.4743	16.0068	6.31E-05	9.57E-04
104	LUC7L	FOXD1	FECH	1.2442	0.4668	16.0041	6.32E-05	9.57E-04
105	BPHL	SPAG4	MRPL12	-1.2065	-0.4516	15.9949	6.35E-05	9.59E-04
106	KLF14	RPS6KB2	MRPL2	-1.3017	-0.5093	15.9876	6.38E-05	9.60E-04
107	MARK2	XRCC5	ZNF347	-1.2638	-0.461	15.9634	6.46E-05	9.62E-04
108	FBXO8	C8orf38	MRPL12	-1.4116	-0.4604	15.9395	6.54E-05	9.63E-04
109	CECR1	ATP6V0D1	FECH	1.285	0.4664	15.898	6.68E-05	9.66E-04
110	RBMX2	USP24	TXLNA	-1.3303	-0.4972	15.8354	6.91E-05	9.73E-04
111	FBXL3	HSD17B1	ZNF347	-1.2884	-0.5171	15.8346	6.91E-05	9.73E-04
112	SPACA1	C1orf181	FECH	-1.2551	-0.464	15.8345	6.91E-05	9.73E-04
113	CHRNA4	FBXW2	MRPL2	1.2065	0.4882	15.8192	6.97E-05	9.76E-04
114	BACE2	NRP2	MRPL2	1.4536	0.5102	15.8144	6.99E-05	9.76E-04
115	DOC1	MOSPD1	FECH	0.9607	0.4763	15.7956	7.06E-05	9.77E-04
116	DNCH1	ELAVL4	TXLNA	-1.129	-0.4592	15.773	7.14E-05	9.79E-04
117	ZNF642	TMEM40	MRPL12	1.149	0.4453	15.7678	7.16E-05	9.79E-04
118	SLC25A20	TIGD6	TXLNA	-1.105	-0.4669	15.7675	7.16E-05	9.79E-04
119	PPP1CB	FBXO24	ZNF347	-1.1921	-0.4712	15.7608	7.19E-05	9.80E-04
120	PHACTR4	ABCF3	MRPL2	0.987	0.4839	15.7134	7.37E-05	9.85E-04

121	PSKH2	RPS10	FECH	-1.2282	-0.5062	15.7018	7.42E-05	9.86E-04
122	NM_001025295	TPD52L2	MRPL2	1.3516	0.5206	15.6018	7.82E-05	9.95E-04
123	COX7A2L	CLPTM1	NKX3-1	1.4259	0.4902	15.5737	7.94E-05	9.99E-04
124	PSKH2	RPS10	ZNF347	-1.3512	-0.4901	15.5725	7.94E-05	9.99E-04

S4 Fig. The area under the receiver operating characteristic (ROC) curve (AUC). As shown, the AUC, sensitivity, and specificity of the classifier are 0.70, 67, and 82, respectively.

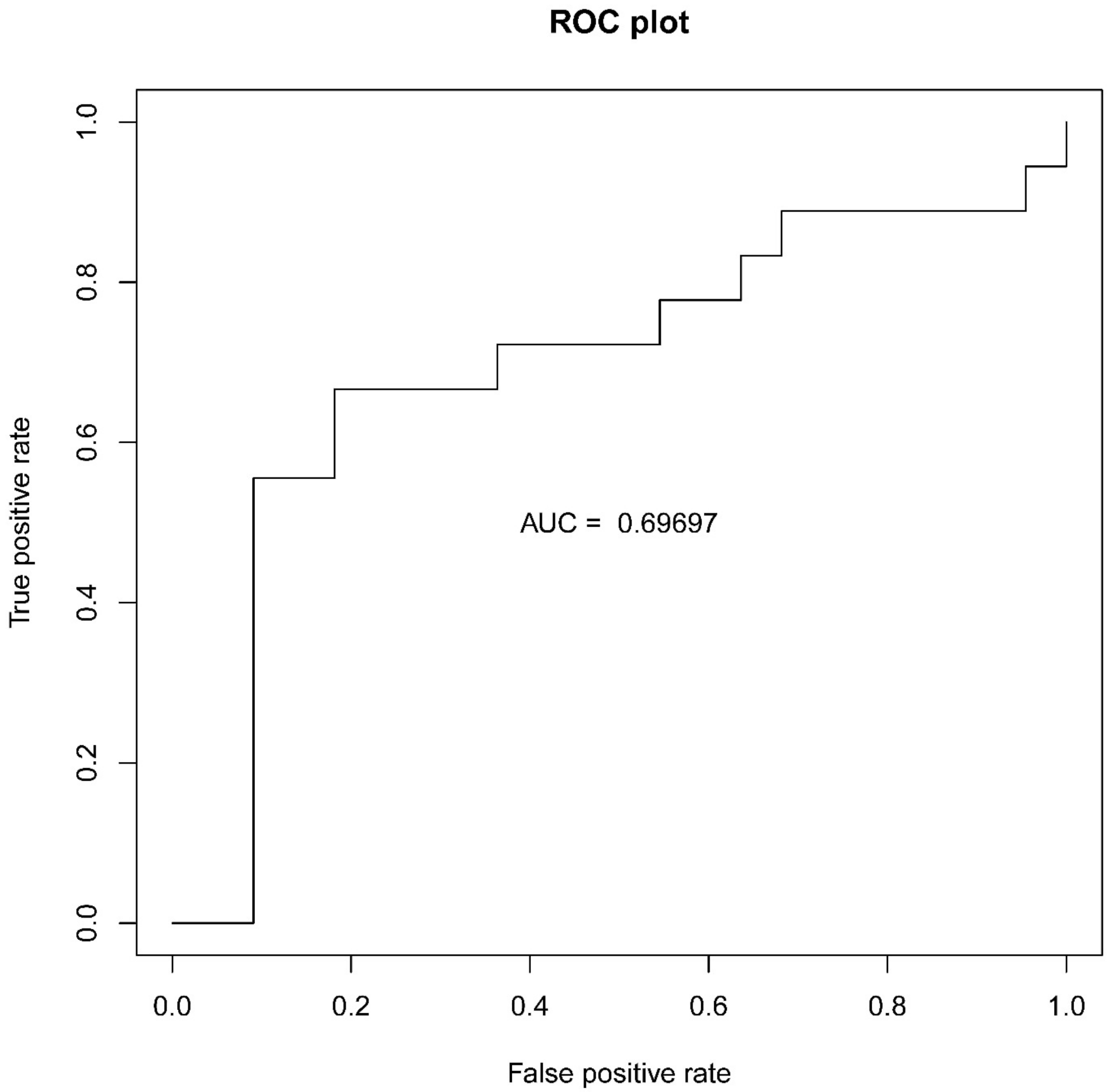

S5 Table. The complete list of enriched Biological Process terms.

GOID	GOTerm	Nr. Genes	Associated Genes	Term PValue	Benjamini Hochberg
GO:0001823	mesonephros development	5	4.424779	7.36E-03	6.62E-02
GO:0031076	embryonic camera-type eye development	3	8.1081085	7.17E-03	6.77E-02
GO:0032481	positive regulation of type I interferon production	4	5.4054055	8.16E-03	7.01E-02
GO:0043401	steroid hormone mediated signaling pathway	7	3.3816426	6.98E-03	7.33E-02
GO:1901989	positive regulation of cell cycle phase transition	4	5.7971015	6.39E-03	7.54E-02
GO:0032462	regulation of protein homooligomerization	3	13.636364	1.60E-03	7.55E-02
GO:0030148	sphingolipid biosynthetic process	4	4.7058825	1.31E-02	8.00E-02
GO:0030261	chromosome condensation	3	7.142857	1.02E-02	8.02E-02
GO:0045931	positive regulation of mitotic cell cycle	5	3.7313433	1.46E-02	8.14E-02
GO:0001756	somitogenesis	4	5.882353	6.07E-03	8.19E-02
GO:0006261	DNA-dependent DNA replication	7	4.5454545	1.36E-03	8.57E-02
GO:0044772	mitotic cell cycle phase transition	12	2.1390374	2.00E-02	9.20E-02
GO:0046426	negative regulation of JAK-STAT cascade	3	5.6603775	1.91E-02	9.25E-02
GO:0030819	positive regulation of cAMP biosynthetic process	4	4.1666665	1.97E-02	9.29E-02
GO:0006415	translational termination	5	4.8076925	5.21E-03	9.84E-02
GO:0014032	neural crest cell development	3	5.172414	2.42E-02	1.04E-01
GO:0048705	skeletal system morphogenesis	6	2.7522936	3.00E-02	1.07E-01
GO:0006352	DNA-templated transcription, initiation	8	3.3755274	4.06E-03	1.10E-01
GO:0006406	mRNA export from nucleus	4	3.539823	3.32E-02	1.14E-01
GO:0034101	erythrocyte homeostasis	4	3.4482758	3.60E-02	1.17E-01
GO:0016072	rRNA metabolic process	7	2.2292993	5.24E-02	1.25E-01
GO:0046128	purine ribonucleoside metabolic process	3	3.8461537	5.12E-02	1.26E-01
GO:0018410	C-terminal protein amino acid modification	3	3.7037036	5.61E-02	1.26E-01
GO:0006397	mRNA processing	10	2.0283976	4.43E-02	1.27E-01
GO:2000106	regulation of leukocyte apoptotic process	3	3.2608695	7.60E-02	1.41E-01
GO:0090630	activation of GTPase activity	3	3.3707864	7.03E-02	1.41E-01
GO:0007051	spindle organization	4	2.6666667	7.76E-02	1.42E-01
GO:0043523	regulation of neuron apoptotic process	5	2.3809524	7.54E-02	1.43E-01
GO:0016573	histone acetylation	4	2.5157232	9.14E-02	1.46E-01
GO:1903902	positive regulation of viral life cycle	3	2.857143	1.03E-01	1.48E-01
GO:1905039	carboxylic acid transmembrane transport	3	2.8301888	1.05E-01	1.49E-01
GO:0098781	ncRNA transcription	3	2.7027028	1.16E-01	1.54E-01
GO:0000725	recombinational repair	3	2.7027028	1.16E-01	1.54E-01
GO:0018107	peptidyl-threonine phosphorylation	3	2.586207	1.28E-01	1.60E-01
GO:0007266	Rho protein signal transduction	4	2.1505377	1.39E-01	1.63E-01
GO:0008286	insulin receptor signaling pathway	3	2.3622048	1.55E-01	1.74E-01
GO:2001257	regulation of cation channel activity	3	2.1126761	1.95E-01	2.02E-01

S6 Table. A GRN subnetwork that includes the regulatory relationship of statistically significant triplets. The regulatory relationship of thirty triplets is detectable in GRN.

Source Node	Target Node	MI
NDUFA6	HIG1	0.13081203
FTSJ3	EIF5A	0.14813939
HIG1	C6orf74	0.1609219
RIOK3	NDUFA6	0.16235824
TPD52L2	SMC2L1	0.18360488
DUSP11	CSNK2A2	0.18492736
MRPL2	DUSP11	0.19274476
DUSP11	ARL5	0.19274476
FECH	FBXL19	0.19274476
ENST00000321394	DNAJA1	0.19274476
TNFSF8	SYNGAP1	0.19274476
TXLNA	RIOK3	0.19274476
LUC7L	FTSJ3	0.19274476
HIG1	GRHPR	0.19274476
NKX3-1	KRTAP11-1	0.19274476
DOC1	C1orf128	0.19274476
GSPT2	ELAVL4	0.19274476
SMC2L1	PTGIR	0.19274476
MRPL12	FBXL3	0.19274476
DNAJC18	CENTB1	0.19676177
VMD2L1	TPD52L2	0.1977407
ZNF347	CP110	0.19833359
SH2D2A	PIN1L	0.2174541
DLG1	CP110	0.22018722
PIPPIN	OR10P1	0.22018722
INPP1	GSPT2	0.22264285
RIOK3	RARA	0.22264285
INPP1	GIPR	0.22297174
CP110	COX7A2L	0.22297174
NKX3-1	INPP1	0.24154852
FECH	DUSP11	0.24154852
VMD2L1	SH2D2A	0.24154852
PIN1L	KLF14	0.24154852
OR10P1	DNAJC18	0.24154852
FECH	ADAMTS10	0.24154852

ADAMTS10	ACIN1	0.24154852
RIOK3	FBXL19	0.24154852
NEUROD2	GDAP1	0.24154852
RIOK3	FOXC2	0.24154852
RARA	PHACTR4	0.24154852
DUSP11	DOC1	0.24445179
VMD2L1	TNFSF8	0.2521945
NFKB2	KLF14	0.25222164
NPM3	ARL5	0.25311404
SH2D2A	PTX1	0.2608839
PTGIR	MRPL12	0.2658702
TXLNA	PIPPIN	0.2704381
NKX3-1	FECH	0.2704381
FBXL19	DLG1	0.2704381
DOC1	CDK9	0.2704381
VMD2L1	PNKP	0.2704381
NPM3	DNCH1	0.2704381
GDAP1	C1orf149	0.27193585
SYMPK	MARK2	0.2768208
PNKP	FOXL1	0.27910915
SYNGAP1	SYMPK	0.2797057
RPS6KB2	PRR5	0.28574502
DUSP11	BACE2	0.29434136
RIOK3	CACNG8	0.29467088
ZNF347	LUC7L	0.30085546
NRN1	MBIP	0.30409884
LRP3	FBXO24	0.30409884
PTGIR	HSD17B1	0.32846805
HIG1	ENST00000321394	0.33070886
INPP1	CDC34	0.3313742
RIOK3	INPP1	0.33450738
ZNF347	RIOK3	0.35228503
TXLNA	RPS6KB2	0.36054718
PIPPIN	OXT	0.3680642
COX7A2L	ACIN1	0.37335092
OXT	MCF2L2	0.37441018
FBXL19	DNASE2	0.37441018
DNASE2	CTRC	0.37441018
PTX1	OXT	0.37441018
OXT	FAM3C	0.37441018
ZNF347	NEUROD2	0.37441018

PRR5	NFKB2	0.38470158
PHACTR4	NPM3	0.41718078
KLF14	CHUK	0.42364937
ZNF347	PHACTR4	0.42853144
ZNF347	VMD2L1	0.49463195
OXT	LRP3	0.49463195
OXT	NRN1	0.7595767
NKX3-1	GPHB5	0.3680642
GPHB5	GDAP1	0.13081203
GDAP1	CKAP5	0.13081203

S7 Table .Gene expression levels of 15 randomly selected genes from a nonfunctioning pituitary adenoma's dataset (Array express accession number: E-TABM-899)

Inv: Invasive pituitary adenoma; Non-Inv: Non-invasive pituitary adenoma; I-IV: grades of pituitary adenoma.

Genes	CBR3	GRIK5	LOXL1	LAMB4	ROPN1	TRPM7	DNAH8	CLPX	SLCO6A1	SLC14A2	AK129904	EGR2	USP16	AARS	ZNF227
Non-Inv-II	-1.17	0.03	-1.45	1.05	-1.17	1.97	0.48	1.05	0.03	1.30	0.03	0.69	0.77	0.03	1.66
Non-Inv-II	-0.69	-0.86	0.41	1.66	-1.30	0.77	0.03	-1.05	-0.22	1.66	0.77	-0.41	-1.97	0.77	-1.05
Non-Inv-II	-0.34	-0.03	1.05	1.30	-0.69	0.22	0.34	-1.66	1.66	-1.66	0.22	-0.28	1.66	1.66	0.48
Non-Inv-II	-1.97	1.05	1.30	-0.09	1.17	0.86	0.95	-0.34	0.28	-0.22	-0.55	0.28	-0.95	0.09	0.09
Inv-IV	-0.03	1.45	1.66	0.48	-0.86	0.03	-1.17	0.15	-0.41	-0.86	-0.34	0.09	-1.30	0.15	-0.55
Inv-IV	0.55	-1.30	-0.34	0.69	1.30	0.15	-0.86	-0.48	1.05	-0.09	0.41	0.03	0.95	-0.77	0.77
Inv-III	-0.86	-0.15	1.17	-0.03	-0.28	1.45	-1.66	-0.22	0.55	1.05	-0.22	-1.45	-1.66	0.55	0.34
Inv-IV	-0.95	0.22	0.34	-0.86	-1.05	0.41	-0.95	0.03	-0.48	0.95	0.48	-0.86	0.22	-1.17	-0.69
Inv-IV	-1.66	-1.17	-0.86	-1.45	-0.95	0.28	-0.09	-0.62	1.45	-1.05	1.66	0.95	-0.22	1.97	0.95
Inv-IV	-0.41	0.28	0.95	0.34	0.09	1.30	0.09	-0.69	0.69	-0.03	0.62	0.86	-0.69	-0.09	-0.62
Inv-III	0.77	-1.05	1.45	-1.17	-0.48	0.95	-0.03	-1.30	1.30	0.77	0.55	1.17	-0.55	-0.41	-1.17
Non-Inv-I	-0.09	-1.97	0.22	-0.15	0.34	0.55	-1.97	0.09	-0.15	-0.48	0.28	-1.97	-0.86	0.62	0.55
Non-Inv-II	0.34	0.55	-1.97	0.03	-0.22	-1.05	1.05	-1.17	-1.97	0.15	-1.05	-1.17	0.34	-0.62	-1.97
Non-Inv-II	-0.28	0.62	-1.17	-0.28	-1.97	1.17	-1.05	0.86	-1.66	0.03	-0.48	-1.66	-1.05	1.30	1.30
Non-Inv-II	-1.45	0.09	-1.66	-0.41	-1.66	0.62	0.41	-1.45	0.09	0.22	1.05	0.55	-0.48	1.17	0.03

Non-Inv-II	-1.30	-1.66	0.48	-0.95	0.62	-0.28	-0.77	-0.77	0.15	0.41	1.45	-0.62	0.48	0.69	1.97
Inv-IV	-0.55	-0.69	-0.22	-1.05	-0.34	0.69	-0.48	-0.86	0.34	0.09	1.17	0.34	0.55	-0.86	-0.28
Non-Inv-II	0.09	0.86	0.55	0.22	0.86	0.34	-0.28	-0.03	0.62	1.17	0.95	1.45	-1.45	0.86	-1.30
Inv-IV	-0.62	-0.41	-0.55	-0.62	-0.77	-0.41	0.62	0.69	-0.09	1.97	-0.41	-0.22	-0.09	-0.15	-0.22
Non-Inv-II	0.48	-0.55	0.28	1.97	-0.09	-0.69	-0.41	0.34	-0.95	0.69	0.15	-0.09	-0.77	0.48	0.15
Inv-IV	0.03	0.41	-1.05	0.09	-0.62	-0.55	-0.62	-0.41	-1.45	0.62	-0.77	-0.48	-0.15	1.45	-1.45
Inv-IV	0.28	-0.77	0.86	-1.66	1.05	0.48	1.30	-0.15	0.95	-0.69	-1.45	1.97	0.41	-0.28	0.69
Inv-IV	-0.15	1.30	-0.48	0.15	0.22	-1.97	-0.34	-0.28	0.48	0.48	0.86	0.48	-0.03	0.95	-0.86
Inv-IV	0.41	1.17	0.62	0.28	0.28	-0.86	0.22	0.28	-0.69	0.34	1.30	1.30	0.03	-0.34	1.17
Inv-III	0.22	0.48	-0.15	1.45	0.55	-0.09	-0.22	-0.55	0.22	0.86	-0.15	-0.69	-0.62	0.41	-0.09
Inv-IV	0.86	0.15	-0.95	0.62	-0.03	-0.15	1.97	0.62	-0.34	-0.95	-0.86	0.77	0.09	-1.66	-0.41
Inv-IV	-1.05	-0.34	0.69	-1.30	-0.55	-0.77	0.28	-1.97	-0.28	-1.30	0.34	0.22	0.69	-0.48	-0.95
Inv-IV	0.62	-0.28	0.77	-0.77	0.95	1.05	-1.45	-0.95	-0.62	-0.62	1.97	-0.95	-1.17	1.05	0.41
Inv-IV	1.30	-1.45	-1.30	0.55	0.15	-0.03	-0.55	0.41	-1.17	-0.34	0.09	-1.30	0.86	-0.03	1.45
Inv-IV	-0.48	1.66	-0.62	-0.48	-0.15	-0.48	-0.15	1.17	-0.86	-0.15	0.69	0.41	0.15	0.34	-0.48
Non-Inv-III	0.15	-0.48	-0.41	-0.69	1.97	-0.34	0.15	0.22	-1.30	-0.41	-0.69	-1.05	0.28	-0.69	-0.34
Non-Inv-III	1.17	0.34	-0.09	-0.55	1.66	-1.17	-1.30	1.97	1.97	0.28	-1.66	1.66	1.17	0.28	0.28
Non-Inv-II	1.45	1.97	-0.28	0.95	0.41	-1.30	0.77	1.66	-0.55	0.55	-0.95	-0.03	0.62	0.22	0.62
Non-Inv-II	0.69	-0.09	0.03	-0.22	-1.45	0.09	0.86	0.55	-1.05	-0.28	-0.62	-0.77	1.97	-0.95	0.86
Inv-III	1.05	0.69	-0.03	-0.34	0.77	-0.95	1.17	0.95	0.77	-0.55	-1.17	0.62	-0.41	-0.22	-0.77
Non-Inv-II	1.66	0.77	1.97	1.17	0.03	-0.62	0.55	-0.09	0.86	-1.45	-1.97	-0.34	-0.34	-1.45	-1.66
Non-Inv-II	1.97	0.95	0.09	0.86	1.45	-1.66	-0.69	0.77	-0.03	-1.97	-1.30	1.05	1.30	-1.30	0.22
Non-Inv-II	0.95	-0.62	-0.69	0.41	0.69	-0.22	1.66	1.45	-0.77	-1.17	-0.03	0.15	1.45	-1.97	-0.15
Inv-III	-0.22	-0.22	0.15	-1.97	-0.41	-1.45	1.45	0.48	1.17	1.45	-0.09	-0.55	1.05	-0.55	1.05
Inv-III	-0.77	-0.95	-0.77	0.77	0.48	1.66	0.69	1.30	0.41	-0.77	-0.28	-0.15	-0.28	-1.05	-0.03

S7 Fig: Analysis of variance for expression levels of 15 exemplary random genes in the different grades of invasive and non-invasive pituitary adenomas.

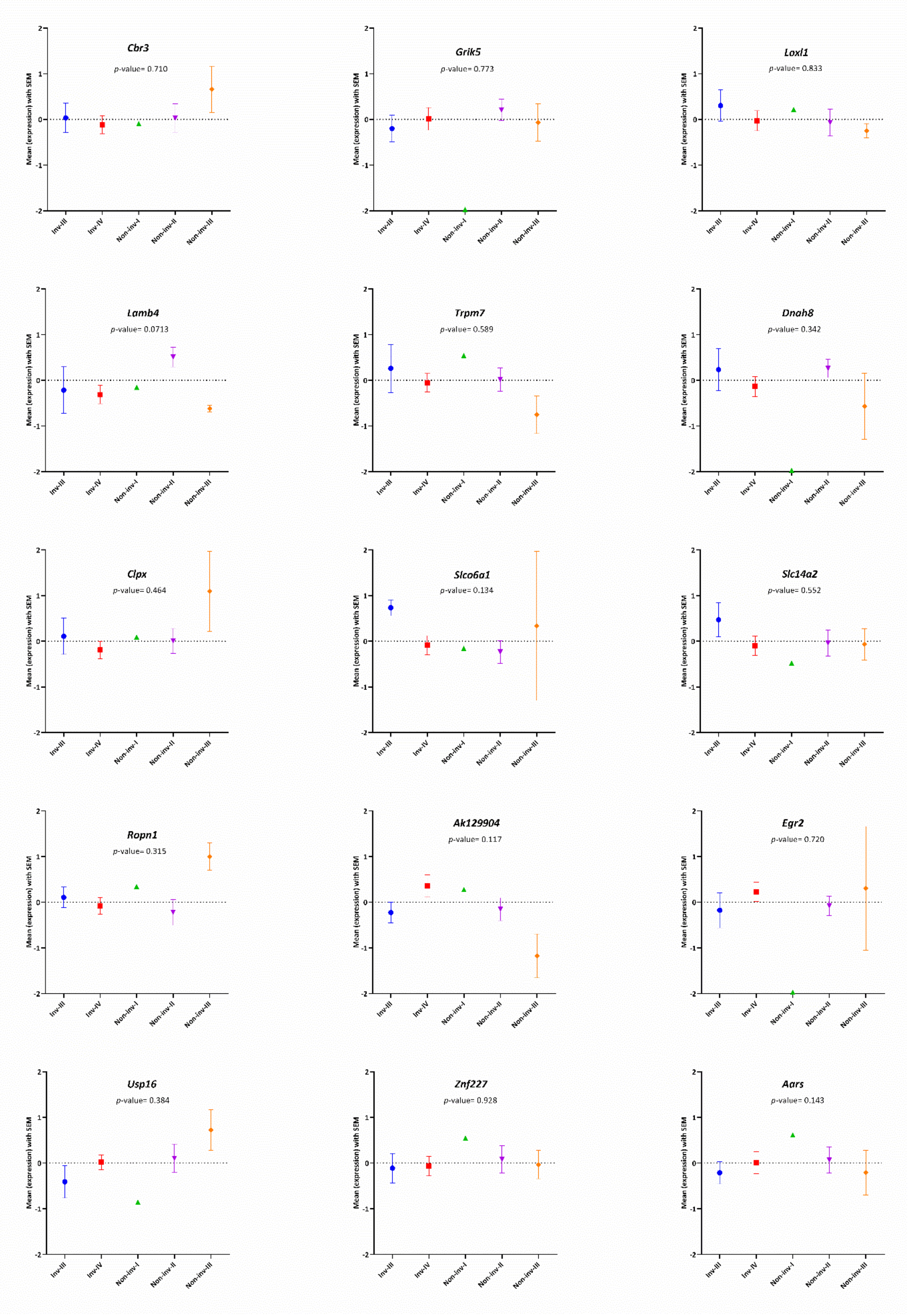
Cbr3
p-value= 0.710
Grik5
p-value= 0.773
Loxl1
p-value= 0.833
Lamb4
p-value= 0.0713
Trpm7
p-value= 0.589
Dnah8
p-value= 0.342
Clpx
p-value= 0.464
Slco6a1
p-value= 0.134
Slc14a2
p-value= 0.552
Ropn1
p-value= 0.315
Ak129904
p-value= 0.117
Egr2
p-value= 0.720
Usp16
p-value= 0.384
Znf227
p-value= 0.928
Aars
p-value= 0.143
Mean (expression) with SEM
Inv-III
Inv-IV
Non-inv-I
Non-inv-II
Non-inv-III

S8 Fig: The association of two identified switch genes with tumor volume, sex, age at surgery and recurrence features.

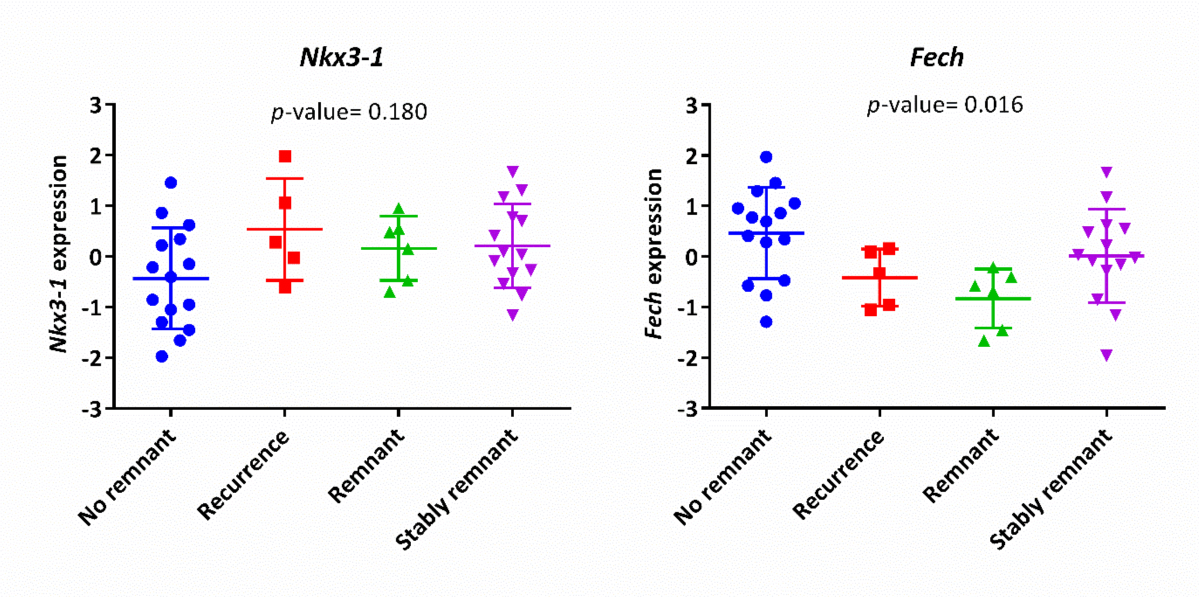

S8-1 Fig: The association of two identified switch genes with recurrence feature. The nonparametric Kruskal–Wallis ANOVA test was used to examine the association of two identified switch genes with recurrence feature. The results indicated significant changes in the expression levels of the *Fech* gene in the different groups of the recurrence feature (*p*-value = 0.016).

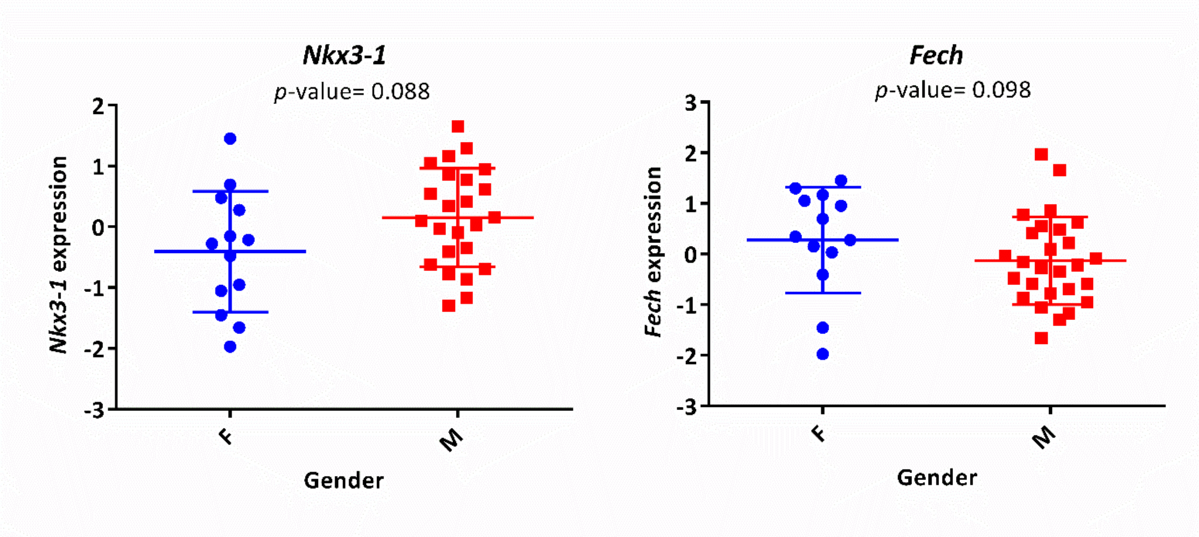

S8-2 Fig: The association of two identified switch genes with gender. The Mann–Whitney test was used for comparisons of gene expression levels between Male (M) and Female (M) groups. The results show that there is not a considerable association between the expression levels of none of the switch genes and gender groups.

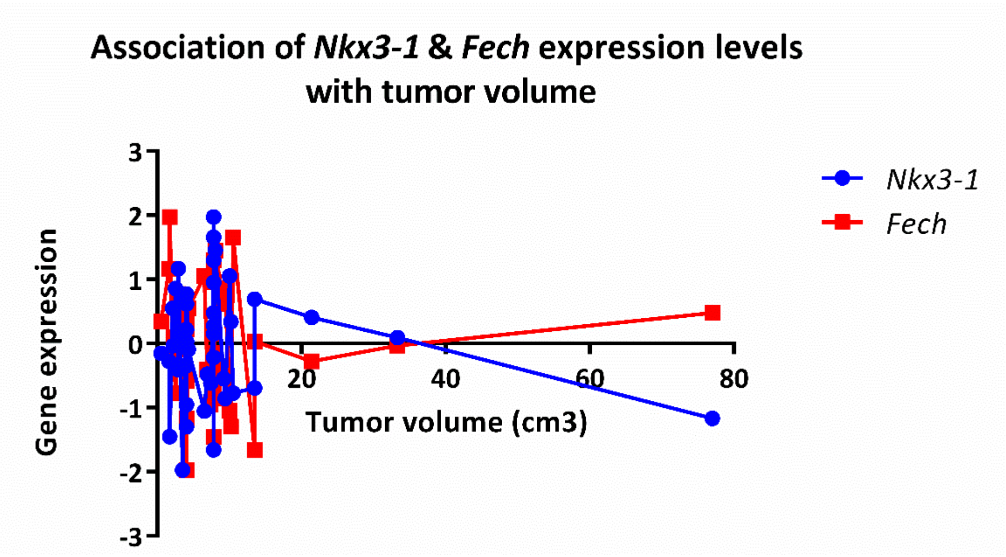

S8-3 Fig: The association of two identified switch genes with tumor volume. The results show that there might be an association between gene expression levels of both switch genes and tumors with volume greater than 20 cm^3. Nevertheless, the number of samples corresponding to the aforementioned tumor is inadequate for such a conclusion (only three samples belong to the tumors greater than 20 cm^3).

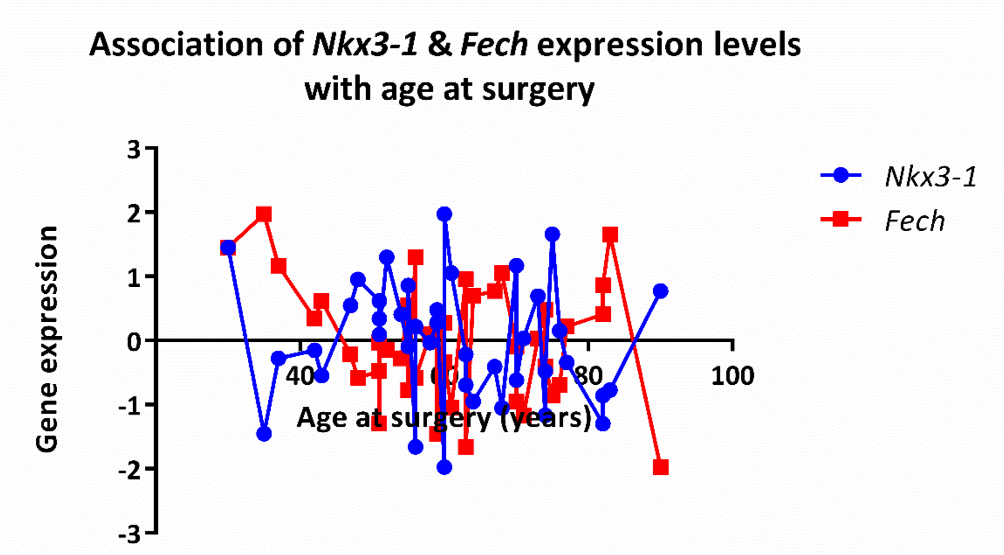

S8-4 Fig: The association of two identified switch genes with age at surgery. As shown in this plot, there is no association between the gene expression levels of switch genes and age at surgery.

S9 Table: Clinical and pathological characteristics of 40 non-functioning pituitary adenomas

Pituitary adenomas (PA) number	Hormonal secretion	Tumor volume (cm3)	Tumor grading	Age at surgery (years)/sex
PA01	NF	10	IV	61/M
PA02	NF	2.2	II	58/M
PA03	NF	77	IV	74/M
PA04	NF	7.8	III	75/M
PA05	NF	33.3	IV	51/M

PA06	NF	21.4	IV	54/M
PA07	NF	4	IV	71/M
PA08	NF	4	IV	90/M
PA09	NF	4	IV	77/M
PA10	NF	10.5	IV	83/M
PA11	NF	2.9	III	70/M
PA12	NF	13.5	IV	73/F
PA13	NF	7.8	IV	52/M
PA14	FSH	7.8	IV	59/F
PA15	NF	7.8	III	59/F
PA16	NF	7.8	IV	76/M
PA17	NF	7.8	III	48/M
PA18	NF	13.5	IV	63/M
PA19	NF	6.9	IV	74/F
PA20	FSH	2.1	IV	47/M
PA25	NF	7.4	III	70/M
PA30	NF	7.8	III	60/M
PA21	NF	2.9	II	67/M
PA22	NF	9.4	II	82/M
PA23	NF	6.5	II	68/F
PA24	NF	3.5	II	60/F
PA26	FSH	9.2	II	43/M
PA27	NF	1.6	II	37/F
PA28	FSH, LH	1.7	II	35/F
PA29	NF	4	II	56/M
PA31	NF	0.5	I	42/F
PA32	NF	4	II	82/M
PA33	NF	4	II	64/F
PA34	NF	7.8	II	63/F
PA35	NF	2.5	II	55/M
PA36	NF	7.8	II	56/F
PA37	NF	10.2	II	51/M
PA38	NF	4	II	51/M
PA39	NF	4.3	II	55/M
PA40	NF	8	II	30/F
